# Vitamin D promotes epithelial tissue repair and host defense responses against influenza H1N1 virus and *Staphylococcus aureus* infections

**DOI:** 10.1186/s12931-023-02477-4

**Published:** 2023-07-05

**Authors:** Shumin Liao, Yanhong Huang, Jinxiu Zhang, Qinglan Xiong, Mengshi Chi, Liang Yang, Junhang Zhang, Liang Li, Yunping Fan

**Affiliations:** 1 Department of Pharmacology, School of Medicine, Southern University of Science and Technology, Shenzhen, China; 2 Department of Otolaryngology, The Seventh Affiliated Hospital of Sun Yat-sen University, Shenzhen, China; 3 Department of Thoracic Surgery, The Seventh Affiliated Hospital of Sun Yat-sen University, Shenzhen, China; 4 Shenzhen Institutes of Advanced Technology, Chinese Academy of Sciences, Shenzhen, China

**Keywords:** Vitamin D, Airway epithelium, Airway organoids, Airway epithelial remodeling, Host responses

## Abstract

**Background:**

Early studies indicated that vitamin D (VD) exerted pleiotropic extra-skeletal effects in the airway, but the definite linkage between VD deficiency and airway host responses remains unclear.

**Methods:**

142 cases of clinical data from Department of Otolaryngology, the Seventh Affiliated Hospital of Sun Yat-sen University, were collected to characterize the relationship between VD deficiency and chronic rhinosinusitis (CRS). Based on the clinical observations, 2.5-D airway epithelial organoids cultured at the air–liquid interface (ALI) were used to simulate the effects of VD treatment in the development of airway epithelium and the modulation of the host responses against influenza H1N1 virus (representing viral infections) and *Staphylococcus aureus* (representing bacterial infections) infections in the airway. The intrinsic mechanisms of VD deficiency underlying epithelial remodeling were mapped by transcriptomic as well as proteomic analyses.

**Results:**

In this study we observed prevailing VD deficiency among inpatients suffering from CRS, a common disease predominantly characterized by epithelial impairment and remodeling. Relative to control organoids cultured without VD, long-term incubation with VD accelerated basal cell proliferation during nasal epithelial development. Under infectious conditions, VD treatment protected the organoids against influenza H1N1 virus and *Staphylococcus aureus* invasions by reinforcing the respiratory host defenses, including upregulation of LL37, suppression (or inhibition) of proinflammatory cytokines, strengthening of epithelial integrity, and mucociliary clearance. In silico analysis of transcriptomics and proteomics suggested that VD modulated the epithelial development and remodeling, involving epithelial cell proliferation/differentiation, epithelial–mesenchymal transition (EMT), and cytokine signaling in the immune system, as well as responses to microbe, cell junction organization, and extracellular matrix organization via PTEN signaling, independent of TGF-β signaling.

**Conclusions:**

Our findings emphasize the importance of managing VD deficiency in clinical settings for the sake of alleviating pathological epithelial remodeling. Vitamin D promotes epithelial tissue repair and host defense responses against influenza H1N1 and *Staphylococcus aureus* infections.

**Supplementary Information:**

The online version contains supplementary material available at 10.1186/s12931-023-02477-4.

## Background

The respiratory epithelium, especially the nasal epithelium, is the first barrier encountered by various detrimental triggers (e.g., noxious pollutants and pathogenic microbes) inhaled into the airway. It behaves as a physical barrier and induces immune responses to maintain the integrity of the airway. Nevertheless, the physical barrier of the epithelium can become leaky in response to defects at cell–cell junctions (especially tight junctions), dysfunction of mucociliary clearance, and imbalanced production of antimicrobial products and proinflammatory cytokines of epithelial cells, eventually resulting in inflammatory and infectious respiratory diseases (e.g., chronic rhinitis) [1–3].

Chronic rhinitis (CRS) is a rather common otolaryngological disease, with worldwide prevalence of 5–12%, and it poses substantial health and economic burdens. Its pathogenesis is complicated and heterogenous, ranging from epithelial impairment and epithelial–mesenchymal transition (EMT) to innate and adaptive immunity dysfunction [1, 2]. Much effort has been exerted to reveal the pathogenesis of CRS; for example, we have noticed the potential linkage between chronic inflammatory airway diseases and VD deficiency [4].

VD deficiency is not a novel concept. Since the early twentieth century, VD-fortified milk has been available to prevent and eradicate rickets [5], although the definition of “VD deficiency” and the clinical significance of VD remain under debate. According to a multiple-population study applying the NIH-led international Vitamin D Standardization Program (VDSP) protocols, approximately 40% of European citizens had serum 25-hydroxyvitamin D (25(OH)D, a very stable metabolite as the major circulating form of VD) concentration of less than 30 nmol/L; if using a threshold of 50 nmol/L, then 44.9, 2.1, and 32.6 million more individuals in Germany, Ireland, and the United Kingdom, respectively, would be classified as VD-deficient [6]. In our study, we brought 142 inpatients with and without chronic nasal inflammatory diseases into analysis and found that approximately 72% of them had VD deficiency at a threshold of 30 ng/mL (75 nmol/L), with 42% of them having a 25(OH)D concentration of less than 20 ng/mL (50 nmol/L).

With clear evidence for the phenomenon of VD deficiency, efforts have been made to elucidate the role of VD in the airway, potentially guiding VD supplementation or food fortification. In addition to its well-established function of balancing bone metabolism and calcium homeostasis, growing evidence is revealing the extra-skeletal properties of VD in the airway. For example, binding of the hormonally bioactive form of VD, 1,25-dihydroxyvitamin D_3_ (1,25(OH)_2_D_3_), to its ligand-dependent receptor induces the transcription of the protein coding gene CAMP (Cathelicidin Antimicrobial Peptide). LL-37, the only human antimicrobial peptide, is produced by epithelial cells from its precursor CAMP; it has a broad antibacterial and antiviral spectrum as well as immunoregulatory function [7]. VD has been reported to be of importance in respiratory immunoregulation, including anti-inflammation and anti-infection, as well as for maintaining epithelial integrity [8, 9]. Nevertheless, some contradictory evidence exists about the anti-infection and anti-inflammation properties of VD. Meanwhile, aberrant epithelial remodeling is a non-negligible issue in such chronic airway disorders as CRS [10], but little is known about VD-associated airway regeneration and remodeling. Therefore, we wished to determine the pleiotropic effects of VD in airway epithelium, focusing on the epithelial regeneration and remodeling (Additional file 1: Fig. S1).

In this present study, we observed the phenomenon of a high incidence of VD deficiency clinically as well as the possibility of communication between VD deficiency and epithelial hyperplasia. We used nasal epithelial organoids, also called airway organoids briefly, to study the impact of VD in the airway, observing acceleration of basal cell proliferation with long-term VD medication and alteration of epithelial host responses against infection by VD. Furthermore, a transcriptomics and proteomics analysis indicated the regulation of epithelial development and remodeling via phosphatase and tensin homolog deleted in chromosome 10 (PTEN) signaling.

## Materials and methods

### Clinical data and nasal epithelial sample collection

Patients diagnosed with chronic rhinosinusitis without nasal polyps (CRSsNP), chronic rhinosinusitis with nasal polyps (CRSwNP), and allergic rhinitis (AR), hospitalized in the Otolaryngology Department of the Seventh Affiliated Hospital of Sun Yat-sen University, were admitted into this study, which ran from December 2020 to February 2021 (winter) and from June to August 2021(summer). Inpatients without airway inflammation were included as healthy control subjects, including those with simple nasal septal deviation, nasal trauma, and sudden deafness. Exclusion criteria included long-course severe systemic diseases (e.g., hypertension and chronic kidney diseases); regular oral or nasal administration of corticosteroids, immunoregulator and antibiotics; and recent airway infection within three months.

Nasal epithelial samples were obtained from patients undergoing functional endoscopic sinus surgery (FESS). The protocol was approved by the ethics committee of the Seventh Affiliated Hospital of Sun Yat-sen University.

### Airway organoid culture

Primary nasal epithelial progenitor cells were isolated from epithelial samples by dissolving in dispase and trypsin. The completely digested tissues were pipetting into flocs and passed through a 70-µm cell strainer to collect the dissociated cells. After centrifugation, the cell pellet was resuspended in PneumaCult Ex-Plus Medium (StemCell Technologies, Canada) and then plated in a collagen (60 µg/mL, Sigma–Aldrich)-coated T-25cm^2^ flask. The medium was changed every 2–3 days. When confluent, the cells were dissolved using accutase and passaged for purification and expansion of progenitor cells. As yielded by the manufacturers, the PneumaCult Ex-Plus Medium can sustain long-term ex vivo expansion of primary epithelial cells for at least 8 passages. Therefore, every passage of all the samples were cryopreserved as biobanks and terminated at passage 5 to ensure differentiation potential. Cells of the passage 3 from 3 donors in our biobanks were resuscitated and expanded in a T25 flask until confluent and then lifted to seed on the tissue culture–treated inserts (pore size: 0.4 µm; Corning, USA) with a concentration of 500,000 cells/mL. The cells in submerged culture were air-lifted in 3–5 days to initiate differentiation and medium in the lower chamber was changed to Pneumacult ALI Medium (StemCell Technologies). An 18-day ALI culture was undertaken to allow cellular differentiation into a pseudostratified mucociliary airway epithelium prior to performing infection experiments. In this study, 3 lines of airway organoids were established from nasal epithelial progenitor cells of 3 donors with CRS.

### VD treatment

To determine the effect of VD on cellular proliferation and differentiation, the bioactive form of VD, 1,25(OH)_2_D_3_, was added into Pneumacult ALI Medium at concentrations of 10 nmol/L (10 nM) and 100 nmol/L (100 nM), from the onset of the ALI culture (denoted as D1) to the end.

To determine the effect of the time and course of VD treatment, 100 nM 1,25(OH)_2_D_3_ was added on ALI culture day 1 (D1, early phase), day 7 (D7, intermediate phase), and one day prior to infection (B1, later phase).

To determine the anti-infection effect of VD on the epithelium, the airway organoids were cultured in medium without VD and then, one day prior to exposure to pathogens, 100 nM 1,25(OH)_2_D_3_ was added. Airway organoids cultured in medium without VD served as the negative control, denoted “ND.”

### Transepithelial electrical resistance (TEER)

At specific time points, the VD-treated airway organoids and the corresponding control cells were brought to room temperature and the medium was changed to fresh medium. After 30 min, TEER measurements were performed using a Millicell-ERS 2 V-Ohmmeter (Millipore, Bedford, MA). According to the guidance of the manufacturer, the TEER was calculated using the following equation:

$${\text{TEER }}\left( {\Omega {\text{ cm}}^{{2}} } \right)\, = \,\left( {R_{{{\text{sample}}}} {-}R_{{{\text{blank}}}} } \right)\, \times \,{\text{effective membrane area }}({\text{cm}}^{{2}} ).$$

The effective membrane area of the inserts used in this study was 0.3 cm^2^, with a diameter of 6.5 mm.

### CCK-8 assay

Airway organoids were cultured in ALI medium in the absence of 1,25(OH)_2_D_3_ for at least 18 days. 10 nM or 100 nM 1,25(OH)_2_D_3_ was added to the medium. After 48 h, the samples were measured using a Cell Counting Kit-8 (CCK-8). CCK-8 regent (100 µL) was added to every fresh medium (900 µL) to form a working solution, which was added to the insert (100 µL) and lower chamber (400 µL) of the airway organoids and incubated for 1.5 h. Inoculums (total volume: 500 µL) were collected and combined for measurement of absorbance at 450 nm.

### *Staphylococcus aureus* (SA) and H1N1 influenza A virus preparation and infection

Cryopreserved SA purchased from the American Type Culture Collection (ATCC 15981) was resuscitated and cultured on a tryptone soybean broth (TSB) agar plate to obtain a single typical colony. One colony was selected with inoculating loop, resuspended in TSB medium, and cultured in a shaker overnight. The next day, prior to infection, the bacteria were subcultured for another 2 h. The prepared bacteria were quantified through spectrophotometry to a standard OD_600_ of 0.5, where the bacterial concentration was approximately 10^8^ cfu/mL.

Influenza H1N1 virus A/PR/8/34 strain was also purchased from ATCC. The propagation of H1N1 was described previously [11]. Briefly, the virus was propagated in special pathogen-free embryonated chicken eggs at 37 °C for 72 h, and the virus-containing allantoic fluid was harvested as a viral stock. Virus titers were determined by the plaque assay via infection of Madin-Darby canine kidney (MDCK) cells, which were 1.25*10^8 pfu/ml. For infection, the airway organoids were inoculated with the virus at a multiplicity of infection (MOI) of 0.01 by diluting the virus stock with *Dulbecco's* Phosphate-Buffered Saline (DPBS).

Both the bacterial and viral inoculums were removed after co-culturing for 2 h. The airway organoids were rinsed three times with DPBS and cultured until the indicated time points.

### Immunochemistry

Airway organoids were fixed with 4% paraformaldehyde (PFA) and then paraffin-embedded after routine dehydration. Sections (5 µm) of the airway organoids were stained with hematoxylin and eosin (H&E) and measured for their immunofluorescence (IF). For IF staining, sections that had been deparaffined and rehydrated were boiled in 10 nM sodium citrate buffer (pH 6.0) at 95 °C for 10 min for antigen retrieval. After rinsing in PBS, sections were blocked in 5% goat serum in PBS (blocking solution) for 1 h at room temperature. Primary antibodies diluted in blocking solution were incubated overnight at 4 °C. Alexa 488- and Alexa 568-labeled secondary antibodies (Invitrogen) were incubated in a dark box at 1:500 dilution in blocking solution for 1 h at room temperature. The slides were mounted in 70% glycerol with DAPI diluted at 1:1500 for simultaneous antifade and nucleus staining. For staining of isolated airway organoids, the cells were spun on slides, fixed with 4% PFA for 10 min, permeabilized with 0.1% Triton X-100 in PBS for 15 min, and then blocked with blocking solution for 1 h at room temperature. Staining for VD receptor (VDR) was performed as described above.

### Quantitative polymerase chain reaction (qPCR)

Total cellular RNA was isolated from the airway organoids using MiniBEST Universal RNA Extraction Kit (Takara, Japan). And viral RNA in the supernatant was lysed and extracted using MiniBEST Viral RNA/DNA Extraction Kit (Takara). The RNA was then reverse transcribed to cDNA using a PrimeScript RT Master Mix kit (Takara). qPCR was performed using Thunderbird SYBR Green qPCR Mix (TOYOBO, Japan) for mRNA quantification. The relative quantification of transcripts of target genes was determined using the comparative cycle threshold against 18s rRNA as an endogenous control, normalized to the control group. To quantify viral replication, standard curve of viral NP gene was determined respectively using the viral stock. Viral gene copy number was calculated based on the Ct value and the standard curve. Primer sequences are listed in Table 1.

**Table 1:** Primer sequences used in this study

Gene	Sequence	
LL37	F	GACACAGCAGTCACCAGAGGAT
	R	TCACAACTGATGTCAAAGGAGCC
VDR	F	GTGGACATCGGCATGATGAAG
	R	GGTCGTAGGTCTTATGGTGGG
RXRa	F	GACGGAGCTTGTGTCCAAGAT
	R	AGTCAGGGTTAAAGAGGACGAT
CYP24A1	F	GATTTTCCGCATGAAGTTGGGT
	R	CCTTCCACGGTTTGATCTCCA
IFNb	F	CTTGGATTCCTACAAAGAAGCAGC
	R	TCCTCCTTCTGGAACTGCTGCA
IL6	F	AGACAGCCACTCACCTCTTCAG
	R	TTCTGCCAGTGCCTCTTTGCTG
IL10	F	TCAAGGCGCATGTGAACTCC
	R	GATGTCAAACTCACTCATGGCT
NP	F	GACGATGCAACGGCTGGTCTG
	R	ACCATTGTTCCAACTCCTTT
Vimentin	F	ATGAAGGAGGAAATGGCTCGTC
	R	GGGTATCAACCAGAGGGAGTGAA
Fibronectin	F	CGGTGGCTGTCAGTCAAAG
	R	AAACCTCGGCTTCCTCCATAA
aSMA	F	AAAAGACAGCTACGTGGGTGA
	R	GCCATGTTCTATCGGGTACTTC
MMP2	F	CCCACTGCGGTTTTCTCGAAT
	R	CAAAGGGGTATCCATCGCCAT
TGF-β	F	CAGCATGGACGTTCGTCTG
	R	AACCACGGTTTGGTCCTTGG
18s rRNA	F	GTAACCCGTTGAACCCCATT
	R	CCATCCAATCGGTAGTAGCG

### Epithelial permeability

4-kDa FITC-dextran (4 mg/mL, 100 µL; Sigma–Aldrich) was added to the apical chamber and fresh medium (500 µL) to the basolateral chamber. After 8 h, the fluorescein level in the basolateral chamber was detected using a microplate reader (Ex/Em = 485/520 nm).

### Ciliary beat frequency (CBF)

Airway organoids were placed under an inverted phase contrast microscope equipped with a high-speed digital video camera. Triplicate video recordings (256f; frame rate: 100 f/s) were made from different fields of view for every set of airway organoids and analyzed using a Sisson-Ammons Video Analysis (SAVA, Ammons Engineering, USA) system for their CBFs.

### RNA sequencing and transcriptomic analysis

RNA sequencing was performed by OE Biotech (Shanghai, China). The total RNA was isolated using the TRIzol reagent (Invitrogen), treated with DNase to deplete the genome DNA. The mRNA was enriched from total RNA by using Oligo (dT) magnetic beads to synthesize cDNA, followed by PCR amplification of the purified cDNA and construction of a cDNA library. The cDNA library was qualified using a Agilent 2100 Bioanalyzer, which ran transcriptome sequencing on Illumina HiSeqTM 2500, producing 150 bp paired-end data. The raw data for each of the libraries from all of the samples were generated and pre-processed using Trimmomatic. The final clean reads were mapped to a human reference genome (GRCh38) using hisat2. The htseq-count tool was used to count the number of reads aligned to protein coding genes. Based on the read counts of every sample, differentially expressed genes (DEGs) were defined, using the DESeq tool to analyze the fold changes (FCs) of the protein coding genes between the samples and the negative binomial distribution (NB) to test the differential expression.

Multiple DEG lists were uploaded to Metascape, a web-based portal providing functional enrichment, interaction analysis, gene annotation, and membership search, leveraging over 40 independent knowledgebases [12]. Lists of DEGs having FCs of greater than or equal to 1.5 and *p* values of less than 0.05 were also submitted to Ingenuity Pathway Analysis (IPA, Qiagen Bioinformatics) software for pathway analysis.

### Liquid chromatography with tandem mass spectrometry (LC–MS/ MS) analysis

Apical secretion of airway organoids was collected with DPBS. Protein in the samples were extracted and digested for LC–MS/ MS analysis. Digested peptides were analyzed by microLC-MS/MS using an UltiMate 3000 RSLC-nano system coupled with an Orbitrap Exploris 480 mass spectrometer (Thermo Fisher Scientific, USA). The MS was operated in data-dependent acquisition mode (DDA). The MS/MS raw data were analyzed by Proteome Discoverer software (Version 2.4 SP1 Thermo Fisher Scientific, USA) with Sequest HT searching engine using a Homo sapiens proteins database downloaded from UniProt website (20,210,903). Only proteins with more than 1.50-fold or less than 0.67-fold change and a p-value < 0.05 in two comparable groups were considered as differential expressed proteins (DEPs). Metascape analysis was used to analyze gene ontology annotation of DEPs [12]. And IPA software was used for pathways and protein–protein interaction network analysis.

### Statistical analysis

All data analysis was expressed as the mean ± SEM. When confirmed the data are normally distributed, the differences between samples were analyzed using unpaired Student’s t test for two groups and two-way ANOVA for multiple comparisons with VD treatment and H1N1/ *Staphylococcus aureus* infection. For clinical data analysis, Chi-square test was used for contingency analysis of the proportion among groups and one-way ANOVA was used for multiple comparisons of every clinical indicator. All tests were two-tailed and statistical significance was quantified as *p < 0.05, **p < 0.01, ***p < 0.001 and ****p < 0.0001. Statistical analysis was performed using GraphPad Prism 9 software.

## Results

### VD deficiency is a highly prevalent subhealth status

A total of 142 cases were enrolled. Additional file 2: Fig. S2 lists the clinical characteristics, categorized by different diseases. Among all the groups, the concentration of serum 25(OH)D_3_, a relatively stable circulating form of VD, was lower than the expected cutoff level 30 ng/mL, fluctuating by 25 ng/mL. Indicators associated with VD, including parathyroid hormone (PTH), calcitonin (CT), and serum calcium and phosphate, were all in normal ranges, as were the peripheral granulocytes, including eosinophils (EOS), basophils (BSO), and neutrophils (NEU). Notably, the level of PTH was mainly distributed close to the high-normal limit, about 50–60 pg/ml; furthermore, the ESO counts in the CRSwNP patients were higher than in the non-inflammatory control with statistical significance (0.33*10^9^/L, p value: 0.0056).

**Fig. 1:**
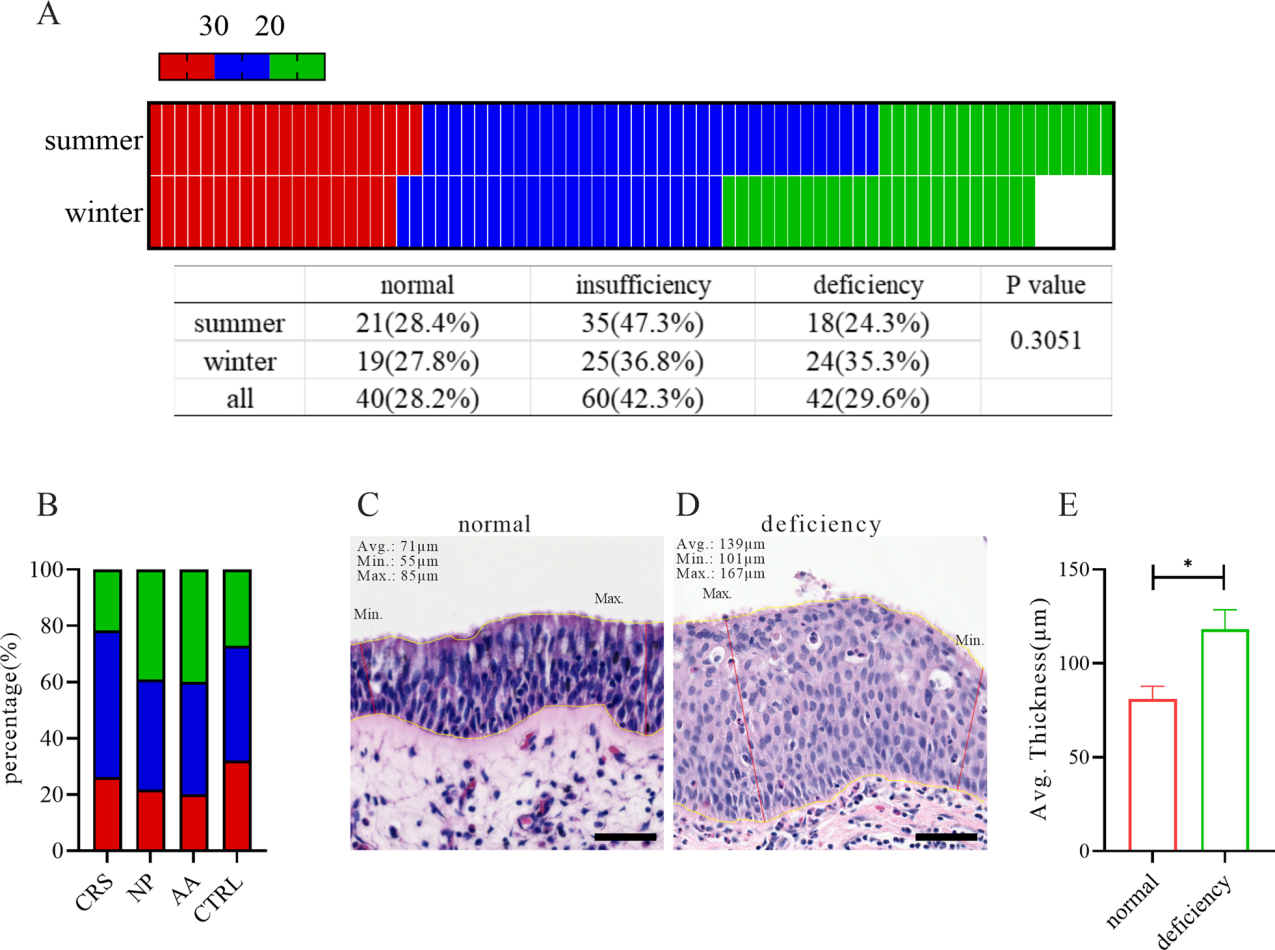
Associated clinical characteristics with VD deficiency.** A** Heatmap of patients’ 25(OH)D_3_ serum levels in summer and winter. Color in red represents patients with normal VD level, blue for VD insufficiency and green for VD deficiency. The table in the lower panel showed the corresponding proportion of the three VD statuses in the two seasons, and chi-square test showed no significant difference (p value = 0.3051).** B** Bar plot of percentage of the three VD statuses in every single disease. **C**, **D** Representative images of nasal epithelium of normal VD and VD deficiency patients, respectively, stained by H&E. Yellow curves traced the basal and apical surfaces of the epithelium to illustrate its thickness, and red lines marked the thinnest and thickest sites, denoted with Min. and Max. The average (Avg.), minimal (Min.) and maximal (Max.) thickness of the epithelium were annotated on the top left corner. Scale bar: 50 μm.** E** Average epithelial thickness of VD deficiency patients was significantly greater than normal VD patients (n = 3 donors, unpaired t test, *: p < 0.05)

To determine the degrees of VD deficiency, we set the cutoff value to be 20 ng/mL; we defined a mild low 25(OH)D_3_ status of 20–30 ng/mL as “VD insufficiency” and a status lower than 20 ng/mL as “VD deficiency.” By this standard, 42.3% of the cases were of VD insufficiency, while 29.6% were of VD deficiency. In total, 72.8% of patients were in a low VD status. The proportions of VD deficiency in the CRSwNP and AR groups were, however, greater, up to 39.13% and 40.00% respectively, indicating a potential linkage between severe VD deficiency and CRSwNP and AR, the inflammatory profiles of which were more like type 2 immune responses (Fig. 1A, B). Notably, the nasal mucosal thickness of CRSwNP patients was greater in those with VD deficiency, averagely 139 μm in VD-deficienct patients versus 71 μm in VD-normal patients, accompanied by basal cell hyperplasia (Fig. 1C, D). According to our analysis, albeit statistically insignificant, the VD deficiency cases increased in winter (Fig. 1A, p value: 0.3051). The distributions of the VD levels in the different disease groups were similar, regardless of whether they had been tested in summer or in winter (Additional file 2: Fig. S2B–D). Scarcely any statistical significance appeared to distinguish the VD insufficiency population from the deficiency population (Additional file 2: Fig. SE, F).

### VD functions in airway organoids, improves epithelial integrity, and induces LL37 production

**Fig. 2:**
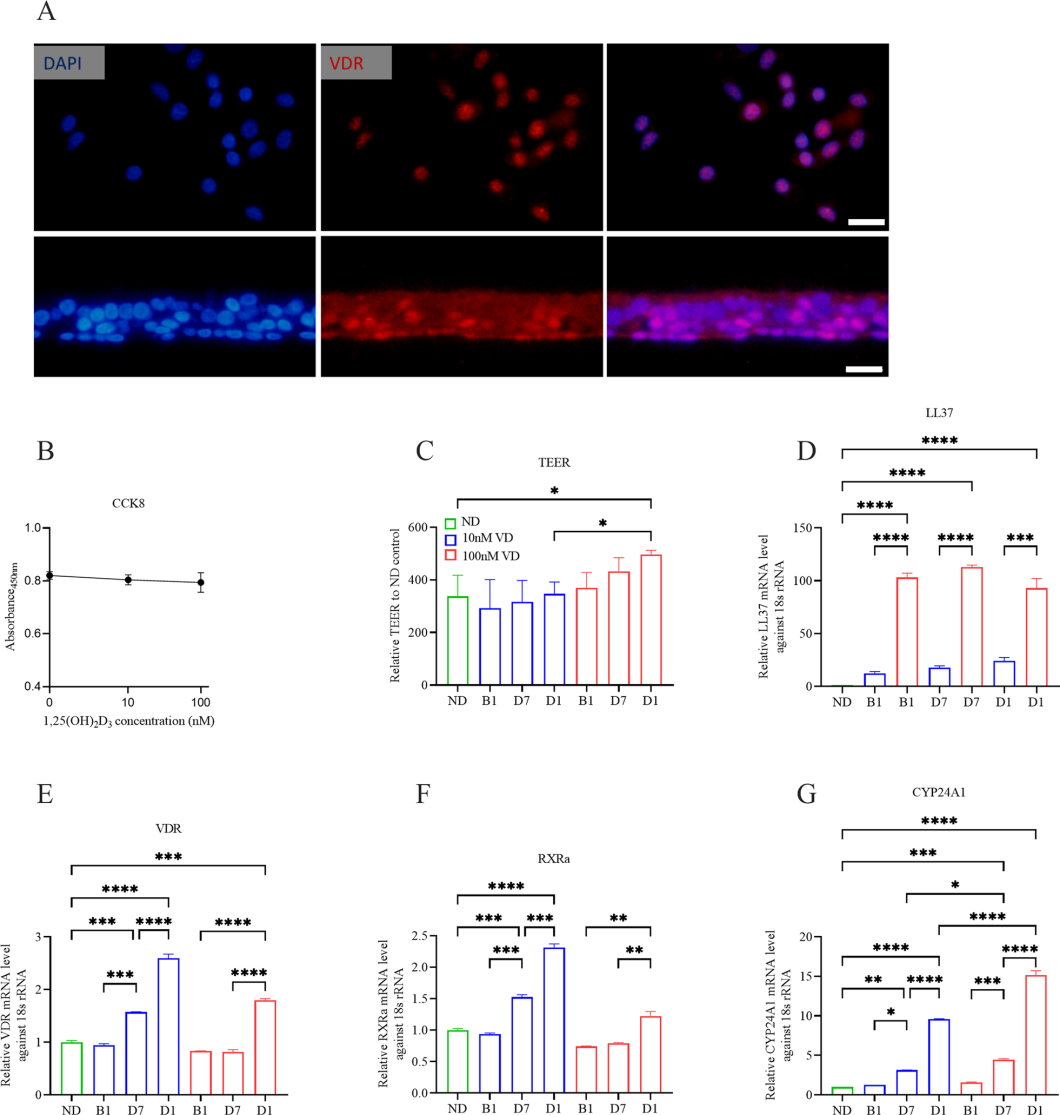
VD functions in airway organoids.** A** Representative immunofluorescence (IF) images of both dissociated and sectioned airway organoids confirmed that airway organoids express VDR (red). Scale bar: 20 μm. **B** CCK8 assay showed no differences in absorbance at 450 nm among airway organoids treated with 0 (control), 10, or 100 nM 1,25(OH)_2_D_3_. **C** Epithelial integrity measured by TEER increased gradually in a treatment course and dose dependent manner. **D**–**G** mRNA levels of LL37, VDR, RXRα and CYP24A1 induced by 1,25(OH)_2_D_3_. High concentration (100 nM) and early phase (D1) treatment of 1,25(OH)_2_D_3_ upregulated the expression of LL37, VDR, RXRα and CYP24A1. Color in red represents airway organoids with 100 nM 1,25(OH)_2_D_3_, blue for 10 nM 1,25(OH)_2_D_3_ and green for ND control

The lipophilic structure of 1,25(OH)_2_D_3_ facilitates the transmembrane movement of the molecule to its high-affinity receptor, specifically VD receptor (VDR). Ubiquitous expression of VDR gene is found in abundant tissues and cell types, including airway epithelial cells and immunocytes [13]. VDR is a nuclear receptor, which is typically heterodimerized with retinoid X receptor (RXR), translocated to nuclei, and bound to the VD response elements (VDRE), thereby regulating the transcription of target genes [8, 13, 14]. To ensure that ex vivo cultured airway organoids responded to 1,25(OH)_2_D_3_, we first detected VDR through IF on both airway organoid sections and isolated epithelial cells, the fluorescence intensity of which was strong in the nuclei and relatively weak in the cytoplasm (Fig. 2A). This result was accordant with the subcellular pattern of VDR in human cell lines shown in *The Human Protein Atlas* (www.proteinatlas.org/ENSG00000111424-VDR/subcellular). Therefore, the airway organoids expressed VDR and were able to respond to exogenous 1,25(OH)_2_D_3_.

Testing for the possible cytotoxicity of high-dose 1,25(OH)_2_D_3_, a CCK8 assay revealed no differences in absorbance at 450 nm among the organoids treated with 0 (control), 10, or 100 nM 1,25(OH)_2_D_3_ (Fig. 2B). Thus, neither 10 nor 100 nM 1,25(OH)_2_D_3_ displayed toxicity toward the airway organoids.

After the airway organoids had differentiated completely, we detected the TEERs of the airway organoids treated with VD in their various culture phases (D1: early phase; D7: intermediate phase; B1: later phase) at the same time. VD treatment tended to increase the TEERs of the airway organoids along the course of treatment and in a dose-dependent manner. Long-term, high-dose VD treatment (D1, 100 nM) resulted in the largest TEER of the airway organoids, indicating robust epithelial integrity (Fig. 2C).

Treatment with both 10 and 100 nM 1,25(OH)_2_D_3_ significantly evoked the expression of LL37, more dramatically in the case of 100 nM 1,25(OH)_2_D_3_, increasing by about 10 folds (Fig. 2D). Long-term, high-dose VD treatment also upregulated the expression of the heterodimers VDR and RXRa and of the 1,25(OH)_2_D_3_ inactivator CYP24A1 (Fig. 2E–G).

### Role of VD in regeneration of airway organoids

We evaluated the effect of pretreatment with VD since the first day of the ALI culture. During the first 11 days, the progenitor cells of airway organoids proliferated dominantly to increase the number of layers of cells, accompanied by gradual differentiation of ciliated (Tubulin +) and goblet (MUC5AC +) cells; in the following week, the airway organoids differentiated to maturity. During the first 11 days, the number of cell layers of the VD-treated airway organoids was greater than that in the ND control group, but the differentiation destinations in both groups were the same, hinting at the proliferation-promoting potential of VD.

**Fig. 3:**
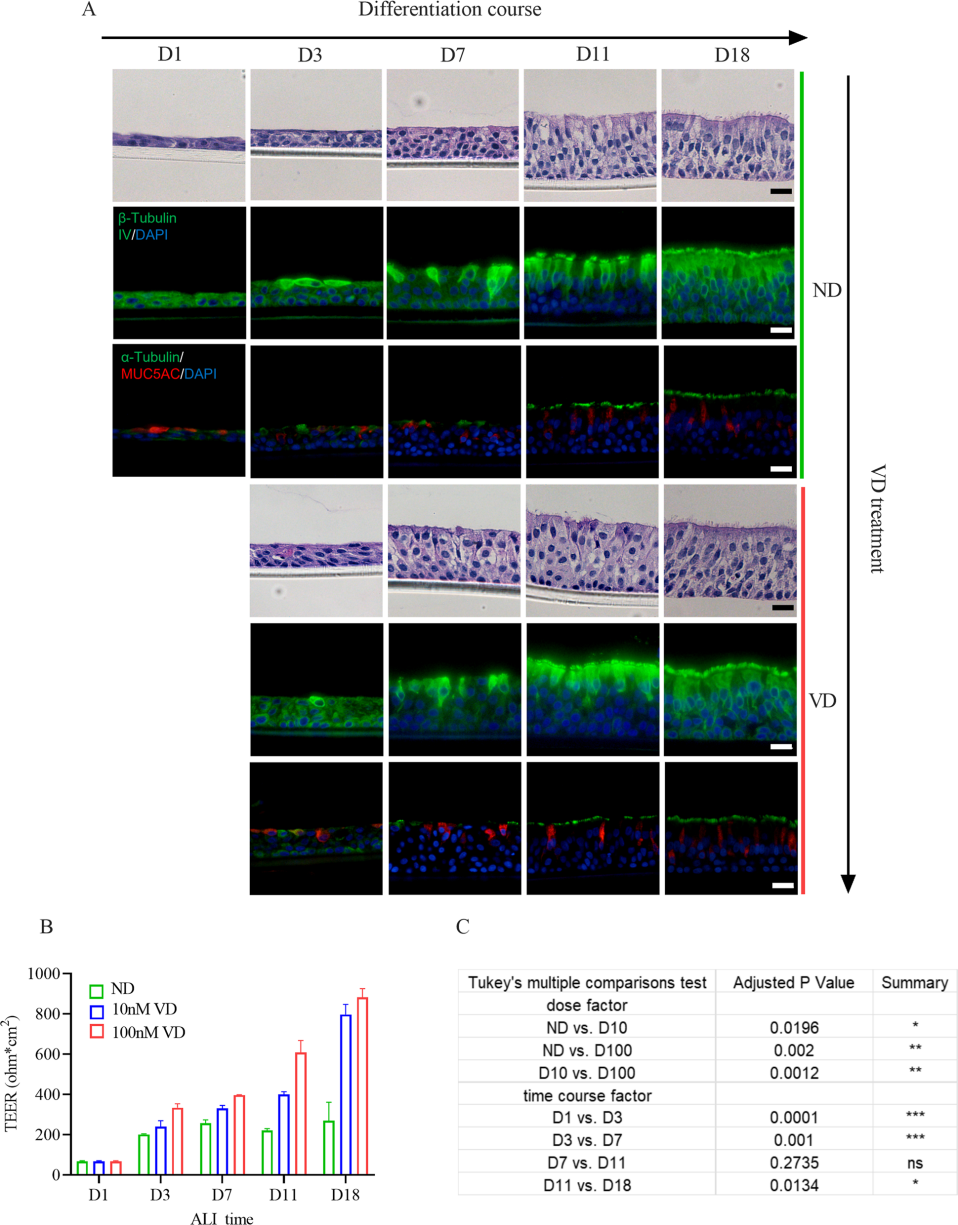
Proliferation and tissue integrity-promoting functions of VD in the generation of airway organoids in vitro.** A** Representative H&E and immunofluorescence images during generation of airway organoids, arranged by differentiation time course in columns and VD treatment in rows. In the first 11 days of ALI culture, cells proliferated rapidly, accompanied with gradual ciliated (marked by β-Tubulin IV) cell differentiation. The cell counts and layer thickness of the epitheliums in VD group increased at a faster pace than those in ND group. In the last week of ALI differentiation (D11-D18), cells differentiated into fully mature airway organoids with quiescent proliferation property. Both black and white scale bars represent 20 μm. **B**, **C** Epithelial integrity of airway organoids at specific ALI differentiation timepoints measured by TEER increased significantly in VD treatment group, especially in 100 nM VD group. ANOVA was performed (n = 3 donors). *: p < 0.05, **: p < 0.01, ***p < 0.001

We measured TEERs to evaluate the development and integrity of the airway organoids. As the regeneration of the airway organoids progressed, the TEER increased in all groups, with the 100 nM 1,25(OH)_2_D_3_ group ranking the highest and the 10 nM 1,25(OH)_2_D_3_ group second. Although the presence of both 10 and 100 nM 1,25(OH)_2_D_3_ strengthened the TEER of the airway organoids, the effect was more powerful at 100 nM (Fig. 3. B).

**Fig. 4:**
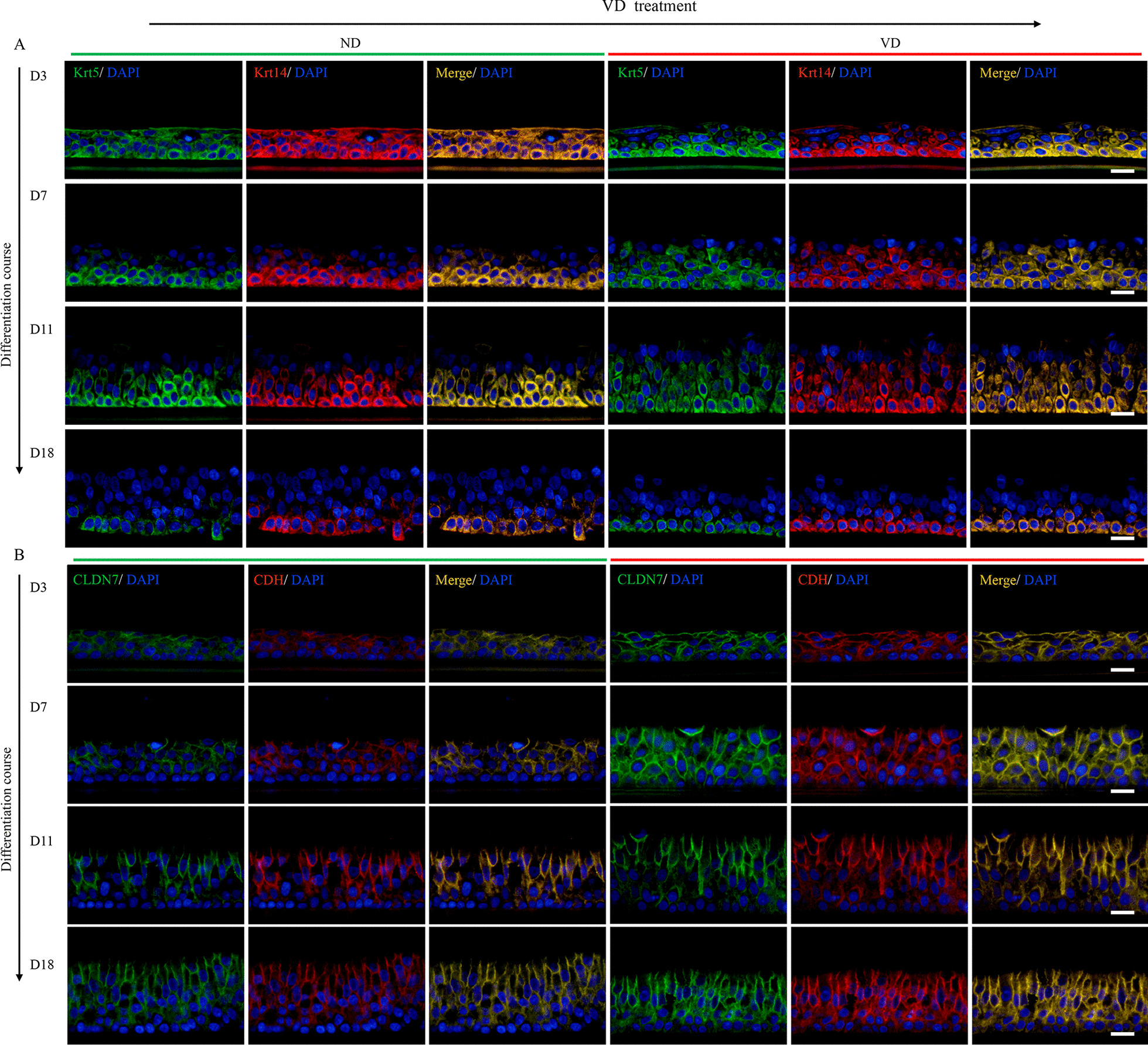
Proliferation-promoting and epithelial integrity-promoting effects of VD in basal cells.** A** IF staining for Krt5/Krt14-positive basal cells, arranged by ALI differentiation time in rows. The left panel was for the ND group and the right panel was for the VD group. In the first 11 days of ALI culture, most cells were Krt5 and Krt14 double positive basal cells. Counts and layer thickness of basal cells in VD group were more than those in the ND group. When fully differentiated at Day18 (D18), only a single layer of basal cells sustained at the base. **B** IF images for tight junction protein claudin 7(CLDN7) and adherence junction protein E-cadherin (CDH), arranged by ALI time in rows. The left panel was for the ND group and the right panel was for VD group. Fluorescent intensity in the VD group were stronger than those in the ND group. Scale bar: 20 μm

**Fig. 5:**
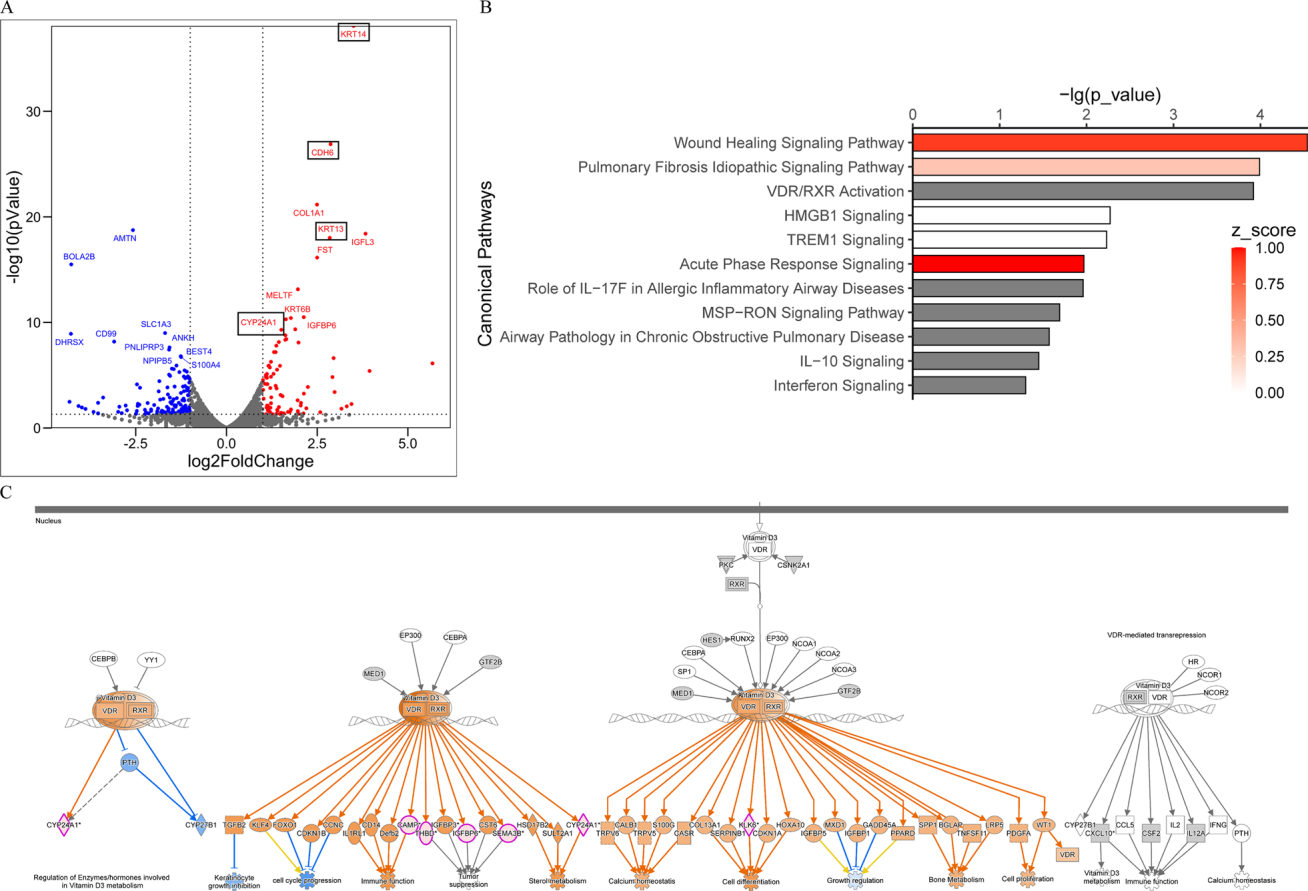
Proliferation-promoting and epithelial integrity-promoting effects of VD in basal cells.** A** Volcano plot for differentially expressed genes (DEGs) of the VD-treated airway organoids. Red dots are up-regulated DEGs and blue dots are down-regulated DEGs, with top 10 DEGs labeled respectively. Among the top 10 up-regulated DEGs, Krt13 and Krt14 are related to cell proliferation and CDH6 is related to adherence junctions. **B** Canonical pathway enrichment analysis of the DEGs in IPA revealed clusters of pathways in airway immunity and host inflammatory responses as well as in epithelial repairing and remodeling, including wound healing signaling pathway and pulmonary fibrosis idiopathic signaling pathway. **C**. The downstream of the VDR/RXR activation involves immune function, cell proliferation and cell differentiation

To clarify the effect of VD on the proliferation and epithelial integrity of the airway organoids, we used IF to trace the progress of basal cells and cell–cell junctions in the course of ALI culture. Basal cells in airway organoids served as progenitor cells; they were capable of both self-renewal and the regeneration of differentiated daughters [15]. During the first 11 days, we found that the airway organoids were predominantly composed of Krt5/Krt14-positive basal cells, with the number of layers and counts of the basal cells in the VD-treated airway organoids (Fig. 4A, right panel D3–D11) being greater than those in the ND control (Fig. 4A, left panel D3–D11). The airway organoids underwent complete differentiation in the following week, with only a single layer of basal cells retained, closely mounted to the basement membrane (Fig. 4A, D18). Similar to the effects on the basal cells, VD treatment enhanced the expression of tight junction protein claudin 7 (CLDN7) and adherence protein E-cadherin (CDH), with the fluorescent intensities of CLDN7 and CDH in the VD-treated airway organoids (Fig. 4B, right panel) being stronger than those in the ND groups (Fig. 4B, left panel). Congruously, transcriptomic profiles of VD-treated airway organoids were characterized by significant upregulation of Krt13, Krt14 and CDH6 gene expressions (Fig. 5A). Pathway analysis revealed that airway immunity, inflammatory host responses, and epithelial repair and remodeling were modulated by VD, as pathways including wound healing signaling pathway, pulmonary fibrosis idiopathic signaling pathway and TREM1 signaling were clustered (Fig. 5B). Meanwhile, the downstream processes of the VDR/RXR activation involved immune function, cell proliferation and cell differentiation (Fig. 5C). Therefore, we conclude that VD promotes basal cell proliferation and strengthens the epithelial integrity in the regeneration of airway organoids. The proliferative properties of organoids augmented by VD here simply mimicked the accelerated development or regeneration of airway epithelium, distinct from the aberrant and multifactorial basal cell hyperplasia in CRS.

### Role of VD in anti-infection and anti-inflammation

**Fig. 6:**
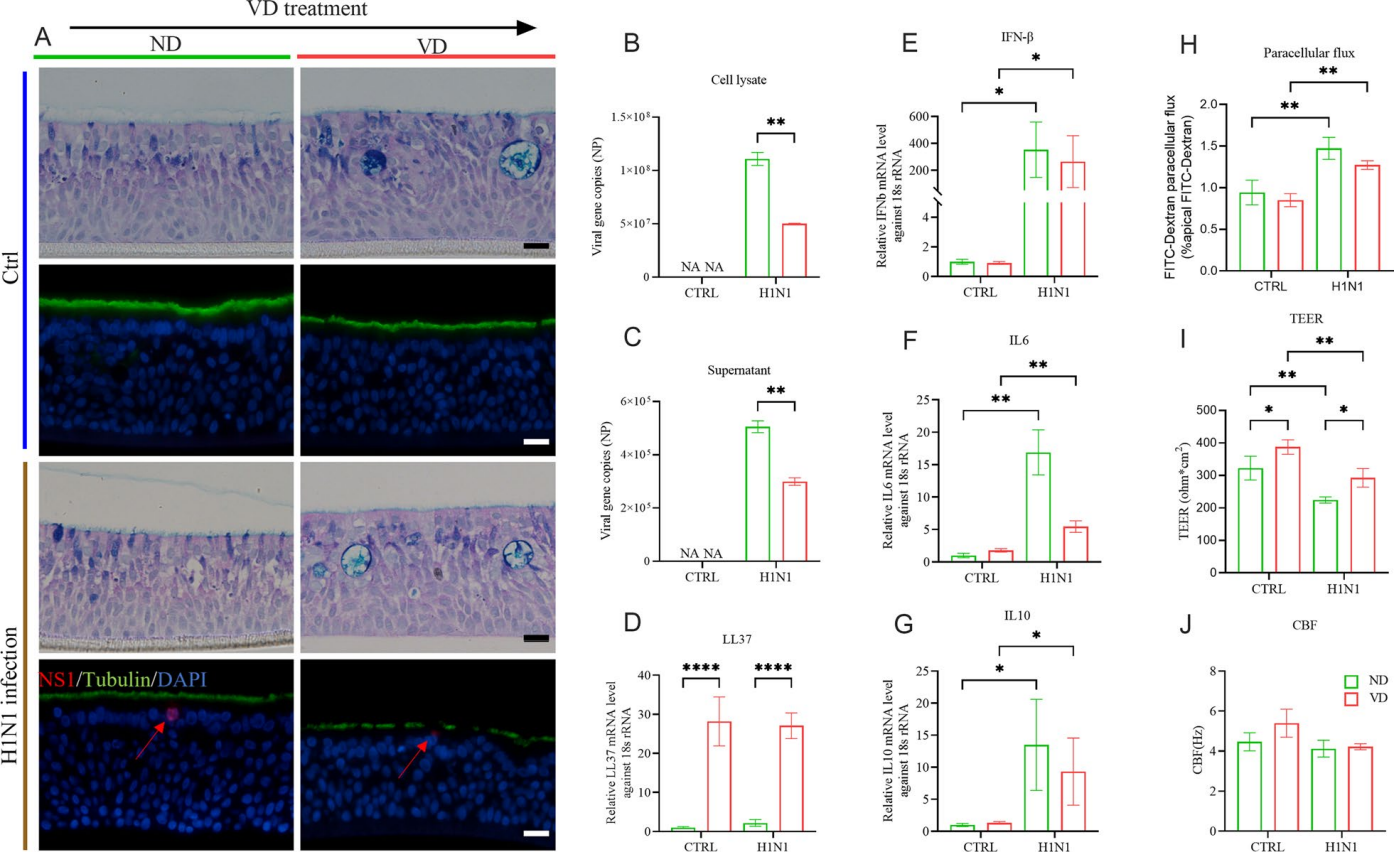
Anti-infection and anti-inflammation effects  of VD in H1N1 infection.** A** Representative Alcian Blue-Periodic Acid Schiff (AB-PAS) and IF staining images for H1N1 infection and the corresponding control in both ND group and VD group. No obvious cell damage or mucus hypersecretion was observed in any group. Scale bar: 20 μm. **B**, **C** The infected organoids (cell lysate) and their supernatants were harvested to detect viral loads by quantifying the copy number of viral nucleoprotein (NP) gene. **D**–**G** mRNA levels of LL37, IFN-β, IL6, and IL10 were shown as relative fold-changes against the control samples. VD treatment significantly upregulated LL37 while downregulated the IL6, IL10 and IFN-β elevation triggered by H1N1 infection. **H** Analysis of cellular junctions of airway organoids via FITC-Dextran paracellular flux quantification. H1N1 infection led to the augmentation of FITC-Dextran paracellular flux, which was apt to ameliorate under the treatment of VD. **I**. Analysis of epithelial integrity and cellular permeability of airway organoids via TEER. VD treatment increased TEER of airway organoids and counteracted the impairment of TEER to airway organoids by H1N1 infection. **J**. Analysis of pathogen clearance of airway organoids via CBF. H1N1 infection disturbed the CBF of airway organoids to interfere the pathogen clearance, while VD was inclined to enhance CBF in opposite effect. Color in green represents ND control and red for 100 nM VD treatment. *: p < 0.05, **: p < 0.01, ***: p < 0.001, *NA* non-available

Although viral replication in airway organoids was detectable, influenza H1N1 virus did not break the integrity of the airway organoids (Fig. 6A). In terms of viral loads, 1,25(OH)_2_D_3_ diminished about 50% of intracellular viral replication (Fig. 6B) as well as 50% of viral release (Fig. 6C). Pretreatment with 1,25(OH)_2_D_3_ significantly induced the expression of LL37, while H1N1 infection did not interfere with the expression of LL37 (Fig. 6D). In the H1N1-infected airway organoids, the mRNA productions of IFN-β, IL-6, and IL-10 were increased significantly by 352.8 folds, 16.9 folds and 13.5 folds, respectively; they tended to be inhibited by VD pretreatment, slightly reduced by 1.3 folds, 3.0 folds and 1.5 folds respectively (Fig. 6E–G). VD pretreatment also tended to maintain the integrity of the airway organoids by improving their cellular junctions, decreasing the paracellular permeability, and accelerating pathogen clearance by increasing the CBF (Fig. 6H–J).

**Fig. 7:**
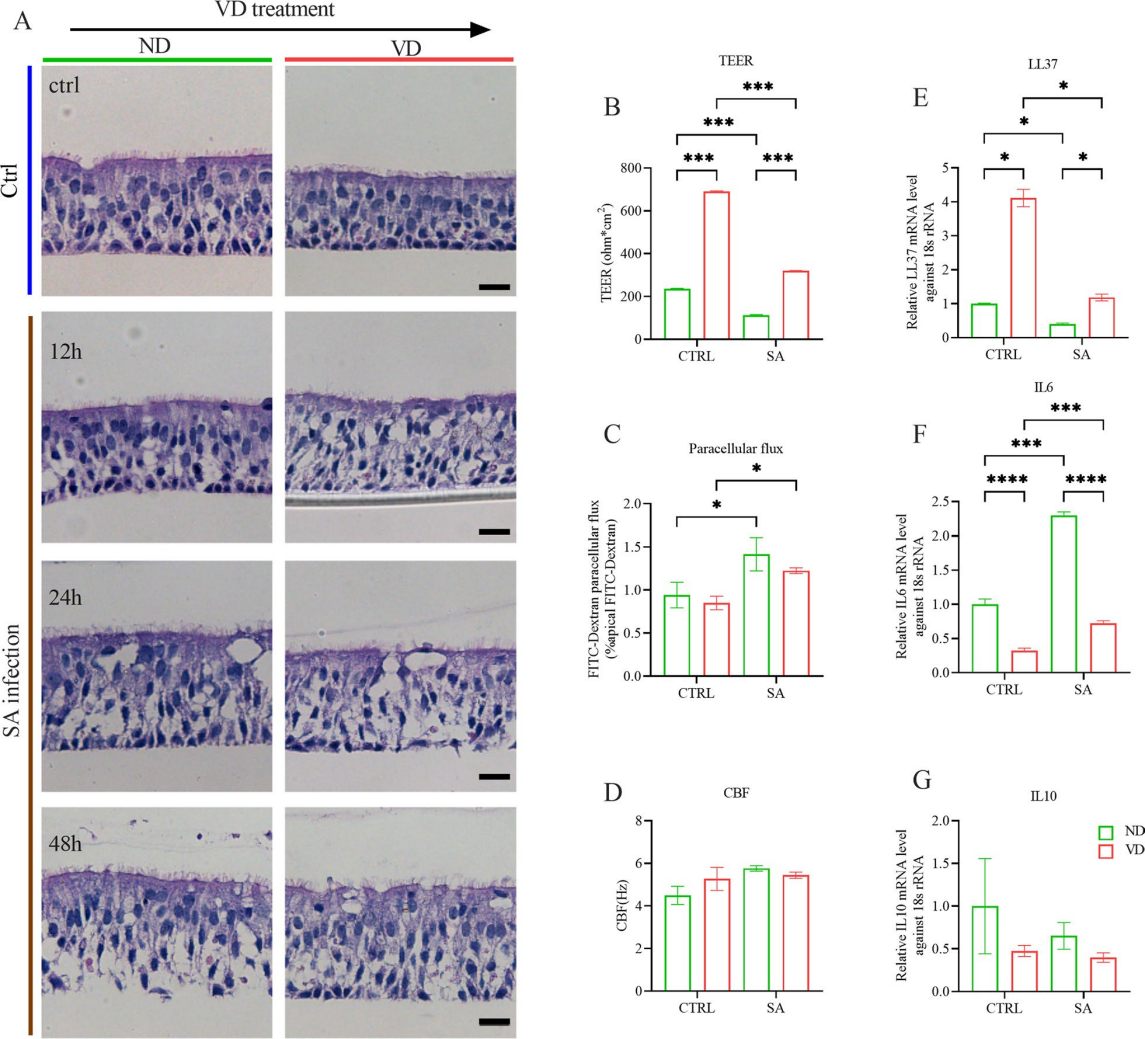
Anti-infection and anti-inflammatory effects of VD in *Staphylococcus aureus* infection.** A** Representative H&E images for SA infection and the corresponding controls in both ND group and VD group. As time prolonged after infection, cellular damage exacerbated, which could be alleviated by VD treatment, especially at 48 h post infection. Scale bar: 20 μm. **B** Analysis of epithelial integrity of airway organoids via TEER. VD treatment increased TEER of airway organoids and counteracted the impairment of TEER to airway organoids by SA infection. **C** Analysis of epithelial permeability of airway organoids via FITC-Dextran paracellular flux. SA infection led to the augmentation of FITC-Dextran paracellular flux, which was apt to ameliorate under the treatment of VD. **D** Analysis of pathogen clearance of airway organoids via CBF. SA infection stimulated the CBF of airway organoids and VD was inclined to enhance CBF as well, both contributing to pathogen clearance. **E**–**G** mRNA levels of LL37, IL6 and IL10 were shown as relative fold-change against control. VD treatment significantly upregulated LL37 and downregulated IL6. Color in green represents ND control and red for 100 nM VD treatment. *: p < 0.05, **: p < 0.01, ***: p < 0.001, ****: p < 0.0001

**Fig. 8:**
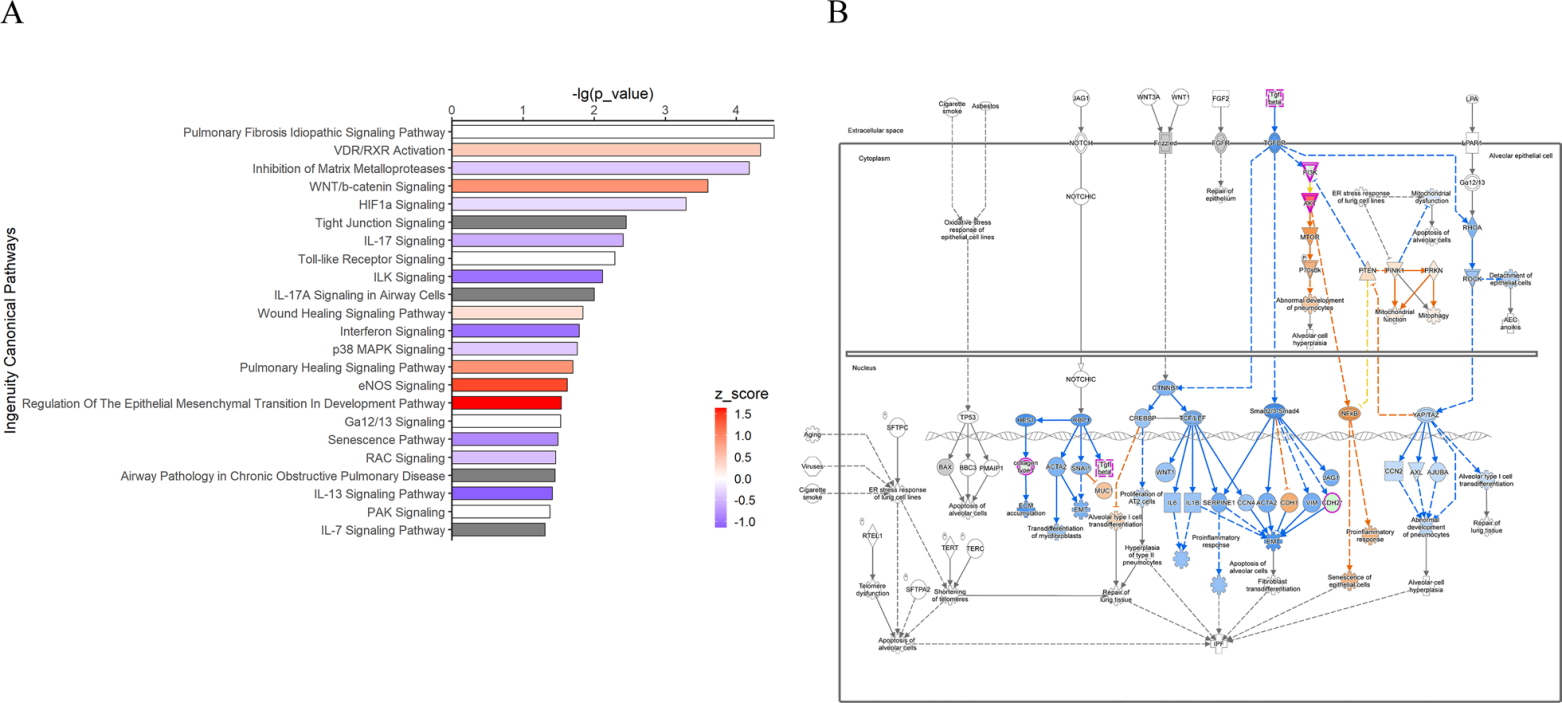
Anti-infection and anti-inflammatory effects of VD in *Staphylococcus aureus* infection. **A** Canonical pathway enrichment analysis of the DEGs in IPA revealed VD treatment contributed to regulation of airway immunity and host inflammatory responses as well as epithelial repairing and remodeling, including wound healing signaling pathway and pulmonary idiopathic signaling pathway, in counteracting SA infection. **B** The downstream of the pulmonary idiopathic signaling pathway regulated by VD involves suppression of EMT, regulation of proinflammatory responses and senescence of epithelial cells

In SA infection experiments, we found that SA destroyed the structure of the airway organoids, with the effect exacerbated as the infection time was prolonged. VD pretreatment alleviated the damage from SA, leading to less broken foci in the VD-treated airway organoids, especially in those harvested at 48 h post infection (hpi) (Fig. 7A). SA infection downregulated the expression of LL37 to 40.1% (p value: 0.0216) and upregulated IL-6 by 2.3 folds (p value: 0.0005), while VD treatment exerted antagonistic effects with 3.0-fold increasement of LL37 (p value: 0.0149) and 68.5% reduction of IL6 (p value: < 0.0001). VD treatment also tended to downregulate the expression of IL-10 (Fig. 7E–G). Although SA infection decreased the TEERs of both the ND and VD groups, the latter maintained the larger TEER by 2.8 folds (p value: 0.0001), suggesting a protective potency of VD (Fig. 7B). VD also had potential effects on maintaining low epithelial permeability upon infection and accelerating the CBF to eliminate pathogens (Fig. 7C, D). The effects of VD in airway damage alleviation and repairment promotion were confirmed by enhanced expression of genes in pathways including pulmonary fibrosis idiopathic signaling pathway, wound healing signaling pathway, inhibition of matrix metalloprotease and tight junction signaling (Fig. 8A). Markedly, the downstream of the pulmonary fibrosis idiopathic signaling pathway led to suppression of EMT, regulation of proinflammatory responses and senescence of epithelial cells, indicating a reduced possibility of fibrosis progression (Fig. 8B).

### Transcriptome profile of VD-treated airway organoids (normalized to ND control): Enrichment in epithelial regeneration/remodeling and epithelial immune regulation

**Fig. 9:**
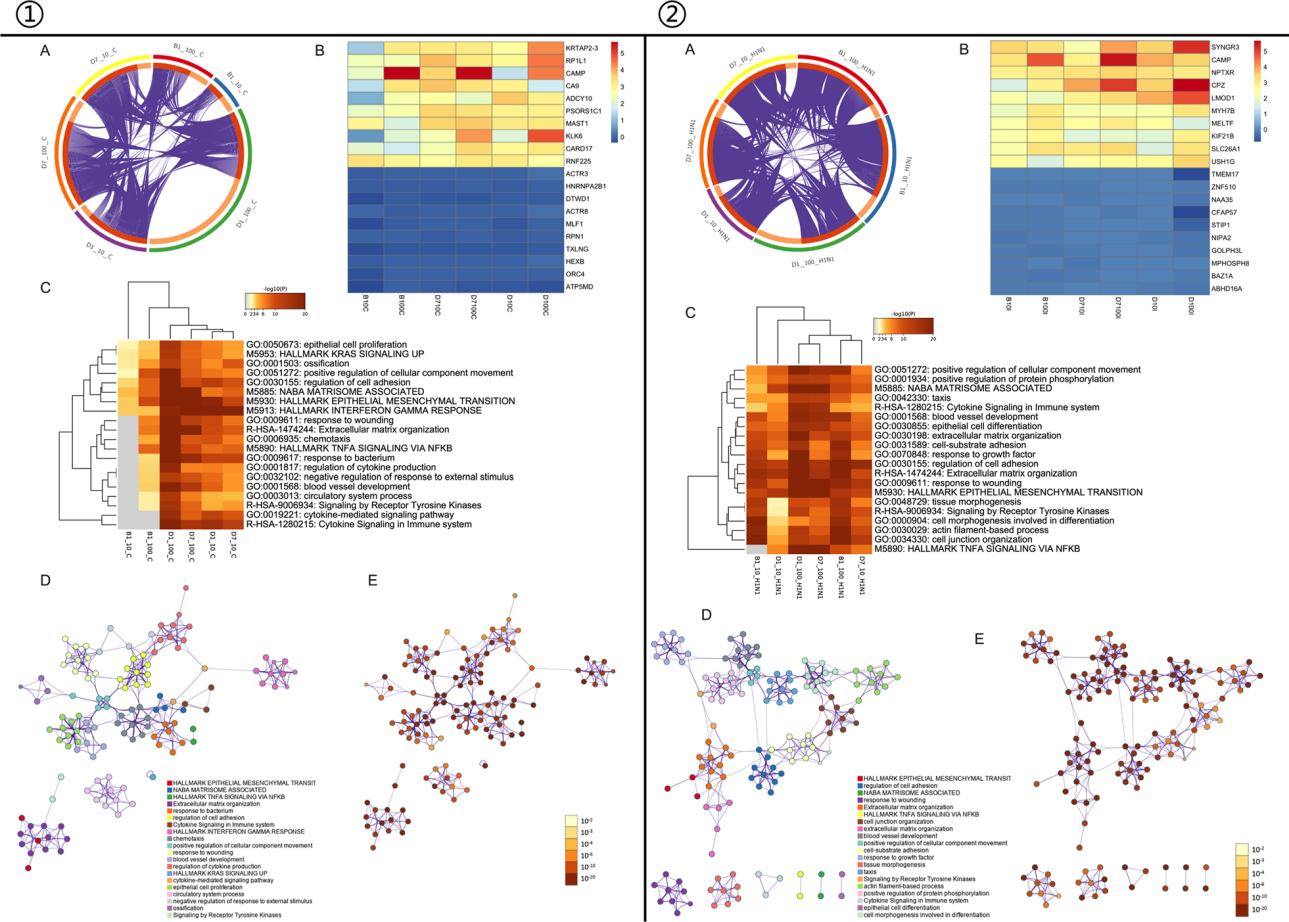
Transcriptomic profiling of VD treated airway organoids.① for transcriptomic profiles of VD-treated non-infected airway organoids normalized to ND control and② for H1N1-infected airway organoids normalized to ND/infection group. **A** Gene overlap analysis (GOA). Each arc on the outside represents the identity of each gene list. Arc on the inside in dark orange color represents the genes that appear in multiple lists and light orange color represents genes that are unique to that gene list. Purple lines link the same gene that are shared by multiple gene lists, showing how gene lists overlap. **B** Heatmap of top 10 upregulated and downregulated DEGs, colored by log transformation of fold change. **C** Heatmap for enrichment analysis based on GO/KEGG database, Reactome pathway database and Molecular signatures database, etc. **D**, **E** A subset of representative terms from the full clusters were selected to convert into a network layout. Terms from the same cluster were colored the same and shared a common term description as label. The same network was also colored according to their p values

Gene overlap analysis on Metascape portal revealed that early phase, high-dose VD treatment (D1_100_C) induced the most DEGs, while later-phase, low-dose VD treatment (B1_10_C) had the least impact (Fig. 9①A). The top 10 upregulated and downregulated DEGs were identified and illustrated as a heatmap, which revealed that VD treatment primarily upregulated gene expression and that the downregulated DEGs varied within a small range of FCs. Cathelicidin antimicrobial peptide (CAMP), the precursor of LL37, was among the top upregulated DEGs. KRTAP2-3 (involved in keratinization), MAST1 (associated with the cytoskeleton), CA9 (involved in cell proliferation and transformation), ADCY10 (associated with ciliary beat regulation), and CARD17 (participating in NOD-like receptor signaling pathway) (Fig. 9B) all contributed significantly to modulation of the host responses of the airway organoids (Fig. 9①B).

We performed hierarchical clustering based on kappa-statistical similarities, with a threshold of 0.3 kappa, to display the enriched terms identified from the GO/KEGG terms, the Reactome pathway database, and the Molecular signatures database. The significant terms identified were all crucial to the host responses, including epithelial cell proliferation, EMT, and cytokine signaling in the immune system (Fig. 9①C). We converted the same subset of representative terms from the full clusters into a network layout, with the enrichment of terms visualized in color. Both the heatmap with a hierarchical tree and the network layout with the *p* values indicated that VD played an important role in the epithelial host responses, including epithelial regeneration/remodeling and epithelial immune regulation (Fig. 9①D–E).

### Transcriptome profile of VD-treated airway organoids infected with H1N1 (normalized to ND infection)

In contrast to simple VD treatment, the combination of VD treatment and H1N1 infection induced comparative DEGs, either unique or overlapped, among the divergent VD pretreatment/H1N1 infection groups (Fig. 9②A). Regulation of DEG expression in this experiment led to a pattern similar to that above for simple VD treatment. Upregulation of DEGs was dominant. CAMP was still one of the top upregulated DEGs (Fig. 9②B). Terms for epithelial cell proliferation, EMT, and cytokine signaling in the immune system were again clustered in the enrichment analysis in this section, emphasizing the importance of VD in epithelial host response regulation, including EMT (Fig. 9②C–E).

### Transcriptional pleiotropic effects of VD and EMT related signaling

**Fig. 10:**
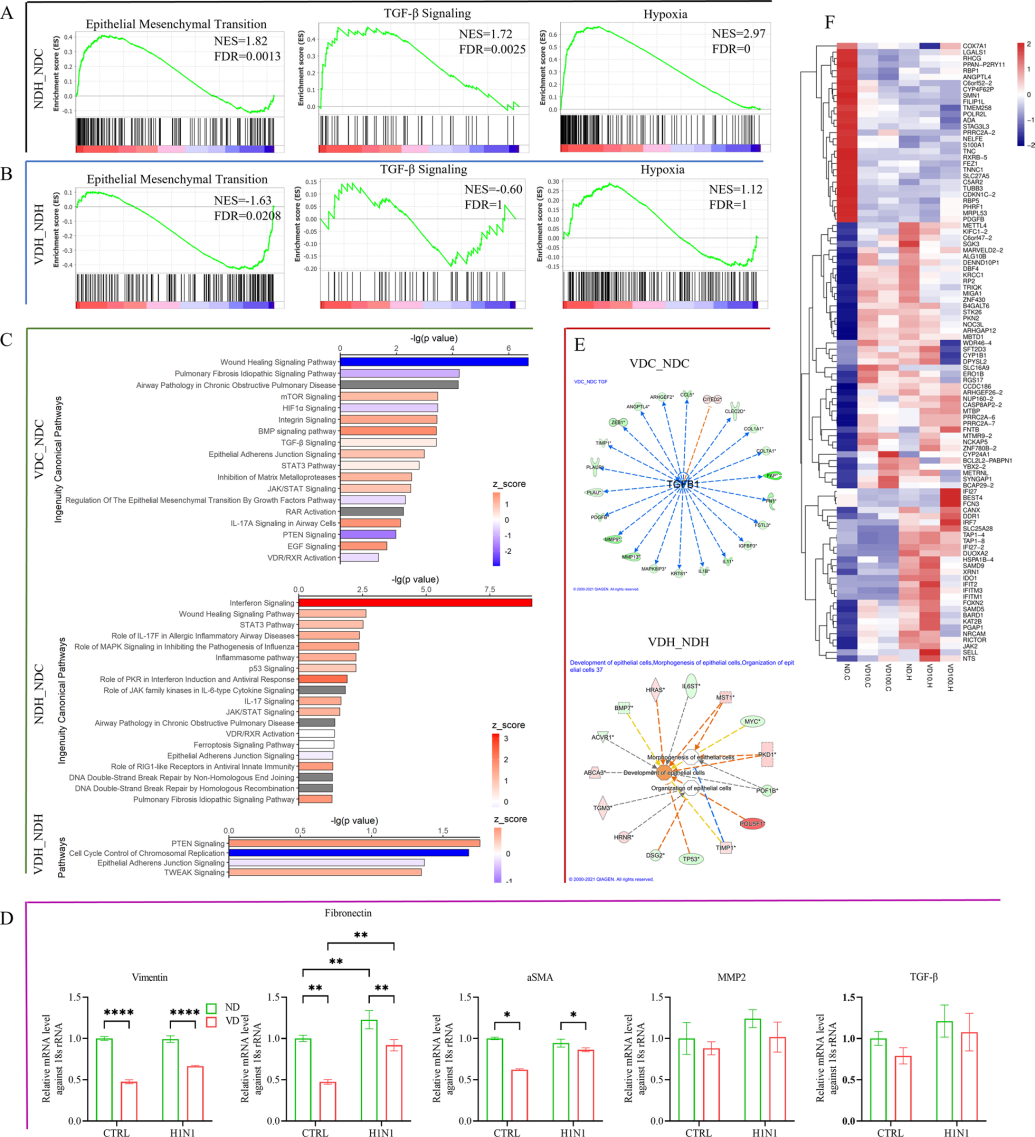
Pleiotropic effects of VD in transcriptome.** A**, **B** Gene set enrichment analysis (GSEA) of transcriptional modulation of H1N1 to airway organoids (NDH_NDC) and of VD treatment in counteracting H1N1 infection (VDH_NDH). **C** Ingenuity Canonical Pathway Analysis (ICPA) in IPA. Both GSEA And ICPA indicated the importance of TGF-β signaling and EMT. **D** Examination of mRNA levels of typical EMT-related genes Vimentin, Fibronectin, α-smooth muscle actin (αSMA), MMP2 and TGF-β. **E**. Upstream regulator analysis in IPA predicted the decreasing TGF-β under VD treatment led to the regulation of other relative genes. Network analysis of VD-modulated DEGs under H1N1 infection clustered in the development, morphogenesis, and organization of epithelial cells. Color in green represents ND control and red for 100 nM VD treatment. **F** Heatmap of top DEGs colored according to their expression values

Gene set enrichment analysis (GSEA) revealed that H1N1 infection activated EMT, TGF-β, and hypoxia signaling. Pretreatment with VD depressed EMT and tended to depress the TGF-β signaling activated by H1N1 infection. In contrast, VD exerted no significant effect on hypoxia (Fig. 10A–B). These results indicate that VD could counteract TGF-β to reverse EMT in a hypoxia-irrelevant pattern.

To further explore the VD-mediated pathways and functions, we ran core analyses of these transcriptional data in IPA software. Ingenuity canonical pathways enrichment revealed that VD treatment modulated multiple pathways, including wound-healing, epithelial adherens junction, inhibition of matrix metalloproteases, regulation of EMT by growth factors, mTOR, TGF-β, PTEN, and EGF signaling. Under H1N1 infection conditions, VD treatment predominantly modulated PTEN signaling (Fig. 10C). According to the upstream regulator analysis in IPA, VD downregulated TGF-β and then TGF-β further regulated relative gene expression as one of the upstream regulators. The VD-modulated DEGs under H1N1 infection were involved in the development, morphogenesis, and organization of epithelial cells (Fig. 10E). Together, these results suggest that VD modulates EMT and epithelial remodeling. Thus, we examined the typical EMT-related genes, including the matrix markers vimentin, fibronectin, and MMP2, and the mesenchymal marker α-smooth muscle actin (αSMA). VD treatment significantly downregulated the expression of vimentin, fibronectin, and αSMA and tended to downregulate MMP2 and TGF-β, revealing the treatment potential of VD in epithelial remodeling via EMT (Fig. 10D).

### Proteomic profiles of VD

To verify the functions of VD indicated at the transcriptional level, we collected the apical secretion of the airway organoids and performed a proteomics analysis. As expected, the enriched ontology clusters of the differential expressed proteins (DEPs) revealed that VD could regulate epithelial development, migration, and response to stimulation (Fig. 11①A). Furthermore, the VD-regulated DEPs were significantly enriched in the canonical pathways, including in the remodeling of epithelial adherens junctions and in the network involving cellular development, growth, and proliferation (Fig. 11①B, C).

**Fig. 11:**
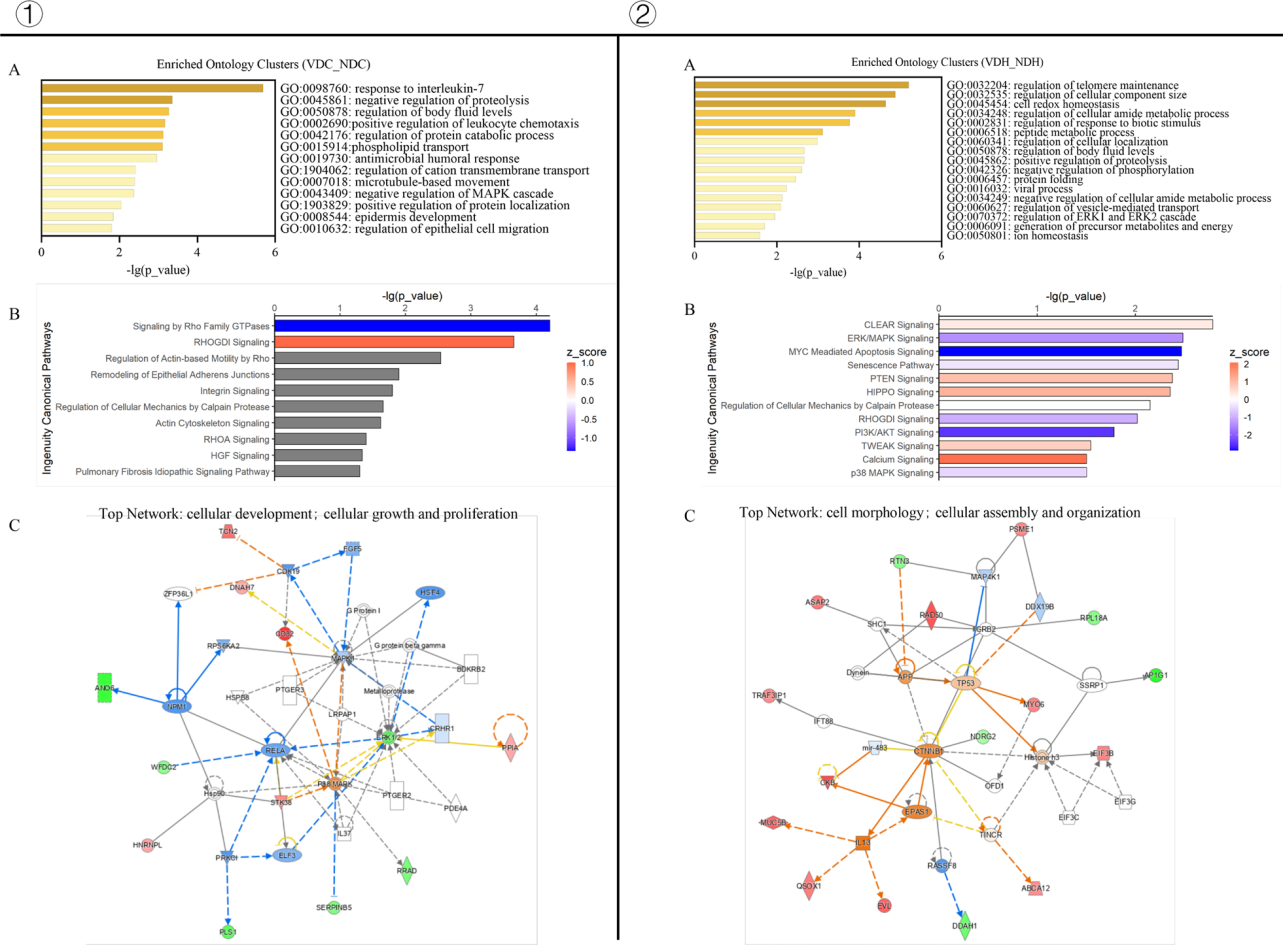
Proteomic profiles of VD.① for proteomic profiles of VD-treated non-infected airway organoids normalized to ND control and ② for H1N1-infected airway organoids normalized to ND/infection group. **A**. Enriched Ontology Clusters in GO database. **B**. Ingenuity Canonical Pathway Analysis (ICPA) in IPA **C**. Network analysis of biological functions of DEPs in IPA

Under H1N1 infection conditions, the VD-regulated DEPs were enriched in the regulation of cellular metabolism (Fig. 11②A). In the canonical pathways, PTEN, HIPPO, and TWEEK signaling were significantly enriched; they were closely related to epithelial proliferation, differentiation, polarization, apoptosis, and remodeling (Fig. 11②B). The protein–protein interaction (PPI) network was associated with cell morphology and cellular assembly and organization (Fig. 11②C). Collectively, these findings demonstrate that VD plays an important role in epithelial development and remodeling.

## Discussion

Discovered as an etiology of endemic rickets a century ago, VD deficiency is an old topic. Many observations and studies have suggested a causative role of VD deficiency in almost all diseases resulting from the extensive cellular sources of VD and VDR and their potential extra-skeletal effects [5, 16]. As early as the 1980s, VD deficiency was identified as a predisposing factor of tuberculosis [17]. A randomized controlled trial in Japan suggested that VD supplementation might help prevent infection of influenza A [18]. Nevertheless, the protective effect of VD in airway infection has been questioned because some studies demonstrated a lack of benefit of VD supplementation [19]. The benefits of VD remain controversial. In this present study, we found a high prevalence of the health status of VD deficiency and, albeit insignificant statistically, a tendency for CRSwNP and AR to be caused by VD deficiency. We examined only a small sample of 142 cases covering a period of only six months, potentially accounting for the negative results of the correlation analysis in the VD deficiency and disease/season associated distribution. A larger-scale cohort study would presumably better elucidate their relationship.

Regarding the ethical limitation and practical complexity for clinical follow-ups, organoid technique as an emerging platform is an ideal alternative for disease modeling. Organoid, self-assembling from stem cells or adult tissue, is a miniature version of an organ that simulates the multicellular architecture and physiological function of the original tissue [20]. In 2009, Hans Clevers et al*.* claimed success in the formation of 3D structures, crypt-villus organoids, derived from Lgr5-possitve adult intestinal stem cells in Matrigel [21], which paced up the rapid advances in organoids. Many organoids have been since then generated to model infectious diseases, Coronavirus Disease 2019 (COVID-19) for instance, providing new insight into human physiology and pathology [20, 22], thus making us adopt airway organoids to uncover the promising impact of VD in airway disorders.

In this study, we verified that VD functions in airway organoids, improves the integrity of airway organoids, and induces the production of LL37; although the current clinical data we collected are limited, they support the significance of VD in airway disorders. We suppose that VD works through long-term modulation of host responses to build up robust protection; it is not a miracle cure. Therefore, we performed an experiment in which we applied various VD treatment courses and doses during ALI culturing of airway organoids. As expected, early-phase and high-dose VD treatment had greatest effect in terms of improvements in the TEERs and the cellular junction expressions of the airway organoids. These finding hint that an adequate course and a sufficient dose are required to ensure that VD works to improve the integrity of the epithelial barrier.

An intact and fully differentiated pseudostratified epithelium is required as a defense against microbial infection. Injury to the epithelium leads to exposure of the susceptible basal cells in the deeper layer, which responds rapidly to injury from a relatively quiescent state, and leads to initiation of EMT in which epithelial cells undergo detachment from one another, proliferation, and reattachment to build up a tight junction. In CRS, cycles of ongoing injury and repair lead to altered states of differentiation and acanthosis with hyperproliferation of basal cells, eventually forming a partially differentiated epithelium [2, 15, 23–26]. In our present study, we traced the progress in the regeneration of the airway organoids at specific times after the onset of ALI culturing. We observed simultaneous proliferation and differentiation during the first 11 days, with proliferation being predominant at this stage. Furthermore, VD treatment in this stage promoted the proliferation of basal cells. Thereafter, differentiation took the leading role, with similar patterns in both the VD treatment and ND control groups. Hence, we conclude that VD accelerates mainly the proliferation of basal cells during regeneration of the airway organoids. Meanwhile, we observed enhancement in the epithelial integrity after VD treatment. The effects of VD treatment on both the promotion of proliferation and the reinforcement of cellular junctions contribute to the regeneration of a healthy epithelium. Interestingly, the proliferation-promoting effect of VD demonstrated in the early stage of regeneration of airway organoids in our study appears to contradict our clinical observation, for the linkage between VD deficiency and epithelial thickening and basal cell hyperplasia. The nasal epithelial thickening and basal cell hyperplasia, however, are the integrated results of multiple factors such as inflammation and infection-induced tissue damage, rather than simply the consequence of VD deficiency whereas the proliferation-promoting effect of VD is more conducive to the repair and reconstruction of airway epithelium. On the other hand, the limitation of organoids narrows their emulation of clinical scenarios. Airway organoids are simply the epithelial compartment of airway, carrying certain extent of genetic and epigenetic features of the patients. By employing the airway epithelial organoids in the study of vitamin D deficiency, the immediate effect of vitamin D deficiency in the infection-induced airway epithelial damage and the associated host responses in epithelial cells can be reflected. However, the main factors causing the epithelial thickening and basal cell hyperplasia in CRS patients with vitamin D deficiency, such as immune cell infiltration and nasal polyp formation, could not be simulated with the commonly used airway epithelial organoids. Future development in organ-on-a-chip that integrates multiple tissues including epithelium, immune cells, fibrotic tissue, and smooth muscle tissue, may provide a better model to represent the long-term effect of vitamin D deficiency in airway infection and regeneration.

The airway epithelium plays a vital role in the pathogenesis of CRS because it serves as the first barrier against detrimental triggers. We found that VD treatment upregulated LL37 expression by the airway organoids dramatically, but also reduced the replication of H1N1 viruses. Furthermore, we found that SA infection inhibited the expression of LL37, which is a mature peptide of hCAP18, cleaved and activated by proteases. Considered as a broad-spectrum endogenous biotic, LL37 is responsible for the elimination of viruses, bacteria, and fungi [27–29]. The upregulation of LL37 indicates the anti-infection potential of VD in airway organoids. And the downregulation of LL37 when infected with SA is consistent with reports that the inflamed loci on the skin of atopic dermatitis with SA infection decreases the capacity of LL37 expression [30], possibly the result of the action of SA against an anti-infection host defense. With VD incubation, the mucociliary clearance (MCC) function of the airway organoids, assessed via CBF, a crucial host defense component, was also inclined to accelerate the clearance of pathogens. At the same time, the increase in epithelial integrity and the decrease in epithelial permeability combine to defend against the invasion of microorganisms. In the context of the anti-inflammation effect of VD, the results are inconsistent. It has been reported that VD can decrease the secretion of proinflammatory cytokines TNF-α, IL-6, and IL-8 [31–33] and increase the level of IL-1β, which is also a proinflammatory cytokine [34]. A report has claimed that the regulation of cytokines by VD is limited to the inhibition of IL-6, with VD exerting no influence on IL-1β, IL-10, and TNF-γ [35]. In our present study, VD tended to suppress the increasing expression of IL-6, IL-10, and IFN-β stimulated by H1N1 and significantly downregulated the expression of IL-6 evoked by SA infection. Collectively, VD does indeed appear to contribute to anti-infection and anti-inflammation in the airway epithelium, but it may serve merely as an adjuvant.

To better understand the detailed mechanism of action of VD in airway host defense, we performed transcriptomics and proteomics analyses that implied that the effects of VD are closely related to epithelial development and remodeling, including epithelial cell proliferation/differentiation, EMT, cytokine signaling in immune system, as well as responses to microbe, cell junction organization, and extracellular matrix organization. The inhibitory effect of VD in EMT have been revealed previously in cancer cells and in pulmonary tissues [36–39]. Furthermore, the serum 25(OH)D concentration has been reported to reversely associate with TGF-β/Smad in COPD patients [40]. Both the findings in the literature and our transcriptomics enrichment data imply that VD can modulate EMT. Furthermore, our GSEA study revealed that VD suppresses EMT signaling activated by H1N1 infection, independent of TGF-β and hypoxia signaling; TGF-β has been considered a typical orchestrator of EMT and hypoxia, as a key initiator of CRS [41, 42]. Therefore, we suppose that VD suppresses epithelial remodeling directly through other pathways. Notably, we found that PTEN signaling was among the significant pathways through which VD modulates to protect airway organoids against H1N1 infection. PTEN, a dual-specificity phosphatase, is well established to be a tumor suppressor, halting cell proliferation, leading to cell cycle arrest, and inducing apoptosis [43]. The loss or overexpression of PTEN appears to participate in cellular senescence, airway remodeling, and SARS-CoV-2 infection [44–47]. Hence, PTEN may play a vital role in the alleviation of epithelial remodeling, probably preventing hyperplasia and promoting maturation, regulated by VD. Nevertheless, we have not performed correlated experiments to investigate how PTEN functions in epithelial remodeling after VD treatment.

## Conclusion

VD promotes nasal epithelial proliferation, differentiation, and epithelial tissue repair, which may be mediated via PTEN signaling. VD also facilitates anti-infection and anti-inflammation, which may be beneficial to ameliorate nasal infection.

## Supplementary Information

**Additional file 1: Figure S1.** Epithelial impairment and remodeling of the nasal mucosa. **A** nasal endoscopy of nasal mucosa. **B** Masson staining of nasal mucosa. Double-headed arrow: epithelial thickening. Yellow arrow: basement membrane thickening. Yellow asterisk: collagen deposition. Red asterisk: goblet cell hyperplasia. Black asterisk: mucus hypersecretion. Black arrow: ciliated cell exfoliation. Blue arrow: basal cell exposure. Red arrow: epithelial exfoliation. Green arrow: epithelial in-growth. Triangle: abnormal structure. Scale bar: 50 μm.

**Additional file 2: Figure S2.** Clinical characteristics of enrolled cases. **A** 142 cases were enrolled and categorized into four groups as CRSsNP, CRSwNP, AR and Ctrl according to their prominent diagnosis, Ctrl group among which was composed of cases of patients without airway diseases as healthy control. Gender proportion, ageas well as cases complicated with AR and AA and cases with cigarette and alcohol consumption were recorded. The average levels of 25D3 were all below the least expected value of 30 ng/ml, either in subgroups or in total. Levels of PTH and CT, which are responsible for homeostasis of Ca and P, were both in normal ranges though levels of PTH were close to the upper limit. Levels of Ca and P were also within normal ranges. Levels of peripheral granulocytes including ESO, BAS and NEU were all among the normal ranges. Analysis of Variancesuggested each laboratory examination of the clinical indicators had no significant difference with the p > 0.05, except that EOS counts in CRSwNP group were higher than those in Ctrl group. **B**–**F** Violin plots of 25D3 concentration of all cases, cases in summer, cases in winter, cases in VD sufficiency and cases in VD deficiency, respectively, grouped by diseases, all showing a similar distribution of VD. *CRSsNP* chronic rhinosinusitis without nasal polyps, *CRSwNP* chronic rhinosinusitis with nasal polyps, *AR* allergic rhinitis, *AA* allergic asthma, *M/f* male/female, *PTH* parathyroid hormone, *CT* calcitonin, *Ca* serum calcium, *P* phosphate, *EOS* eosinophils, *BAS* basophils, *NEU* neutrophils.

## Data Availability

The transcriptomic data have been deposited in NCBI's Gene Expression Omnibus (Edgar et al., 2002) and are accessible through GEO Series accession number GSE224094 (https://www.ncbi.nlm.nih.gov/geo/query/acc.cgi?acc = GSE224094). The mass spectrometry proteomics data have been deposited to the ProteomeXchange Consortium (http://proteomecentral.proteomexchange.org) via the iProX partner repository [1] with the dataset identifier PXD031956.

## Abbreviations

VD: Vitamin D
VDR: Vitamin D receptor
CRS: Chronic rhinosinusitis
CRSsNP: Chronic rhinosinusitis without nasal polyps
CRSwNP: Chronic rhinosinusitis with nasal polyps
AR: Allergic rhinitis
AA: Allergic asthma
PTH: Parathyroid hormone
CT: Calcitonin
Ca: Serum calcium
P: Phosphate
EOS: Eosinophils
BAS: Basophils
NEU: Neutrophils
EMT: Epithelial-mesenchymal transition
CAMP: Cathelicidin antimicrobial peptide
SA: *Staphylococcus aureus*
ALI: Air–liquid interface
CCK8: Cell count kit 8
TEER: Transepithelial electrical resistance
CBF: Ciliary beating frenquency
GSEA: Gene set enrichment analysis
PTEN: Phosphatase and tensin homolog deleted in chromosome 10
