# Supplementary figures and images for: Vitamin D promotes epithelial tissue repair and host defense responses against influenza H1N1 virus and Staphylococcus aureus infections
Source: Respir Res. 2023 Jul 5;24:175. doi: 10.1186/s12931-023-02477-4 (PMC10324174; doi:10.1186/s12931-023-02477-4)

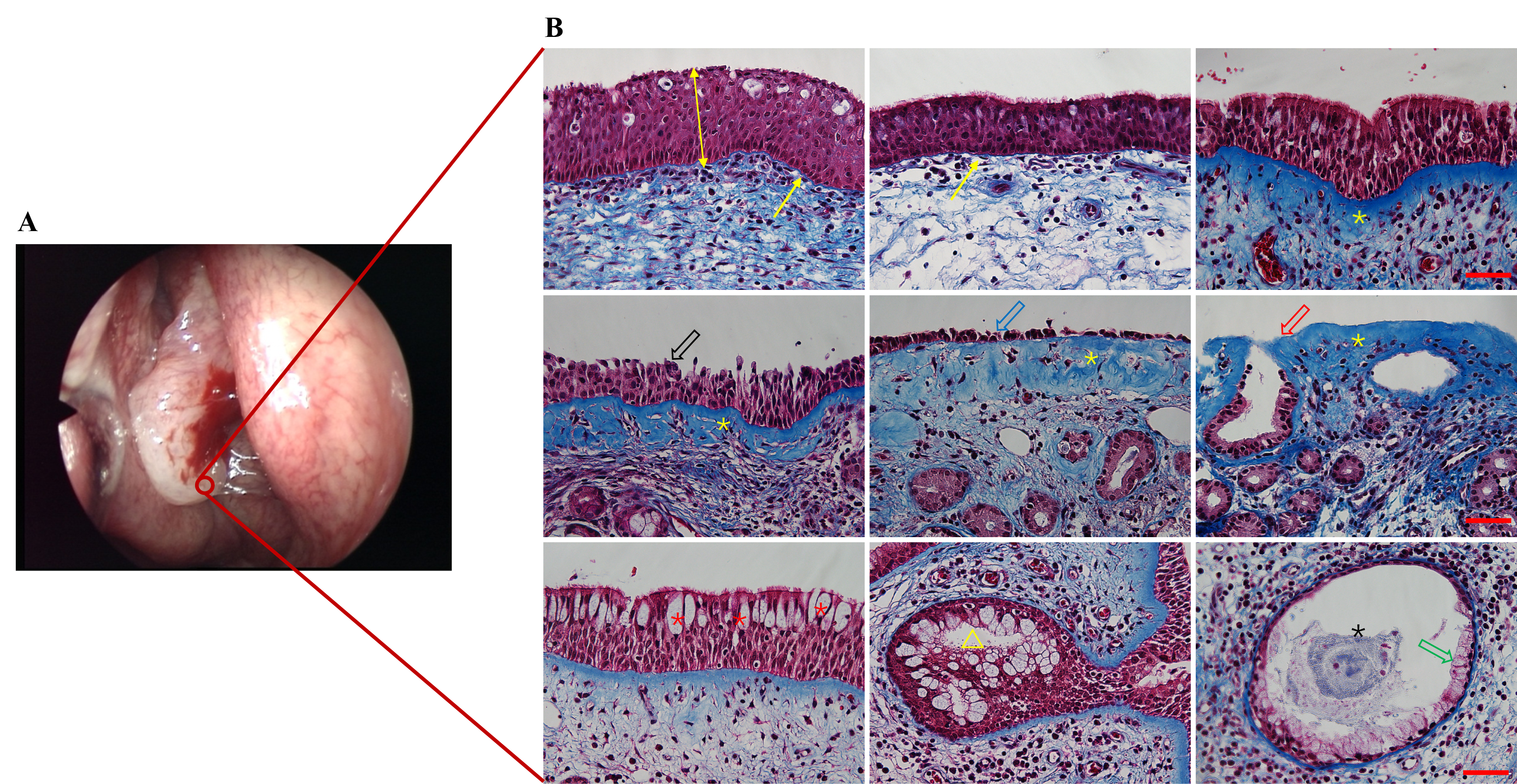

Supplement: Supplementary file 1 — Additional file 1: Figure S1. Epithelial impairment and remodeling of the nasal mucosa. A nasal endoscopy of nasal mucosa. B Masson staining of nasal mucosa. Double-headed arrow: epithelial thickening. Yellow arrow: basement membrane thickening. Yellow asterisk: collagen deposition. Red asterisk: goblet cell hyperplasia. Black asterisk: mucus hypersecretion. Black arrow: ciliated cell exfoliation. Blue arrow: basal cell exposure. Red arrow: epithelial exfoliation. Green arrow: epithelial in-growth. Triangle: abnormal structure. Scale bar: 50 μm. [file 12931_2023_2477_MOESM1_ESM.tif]

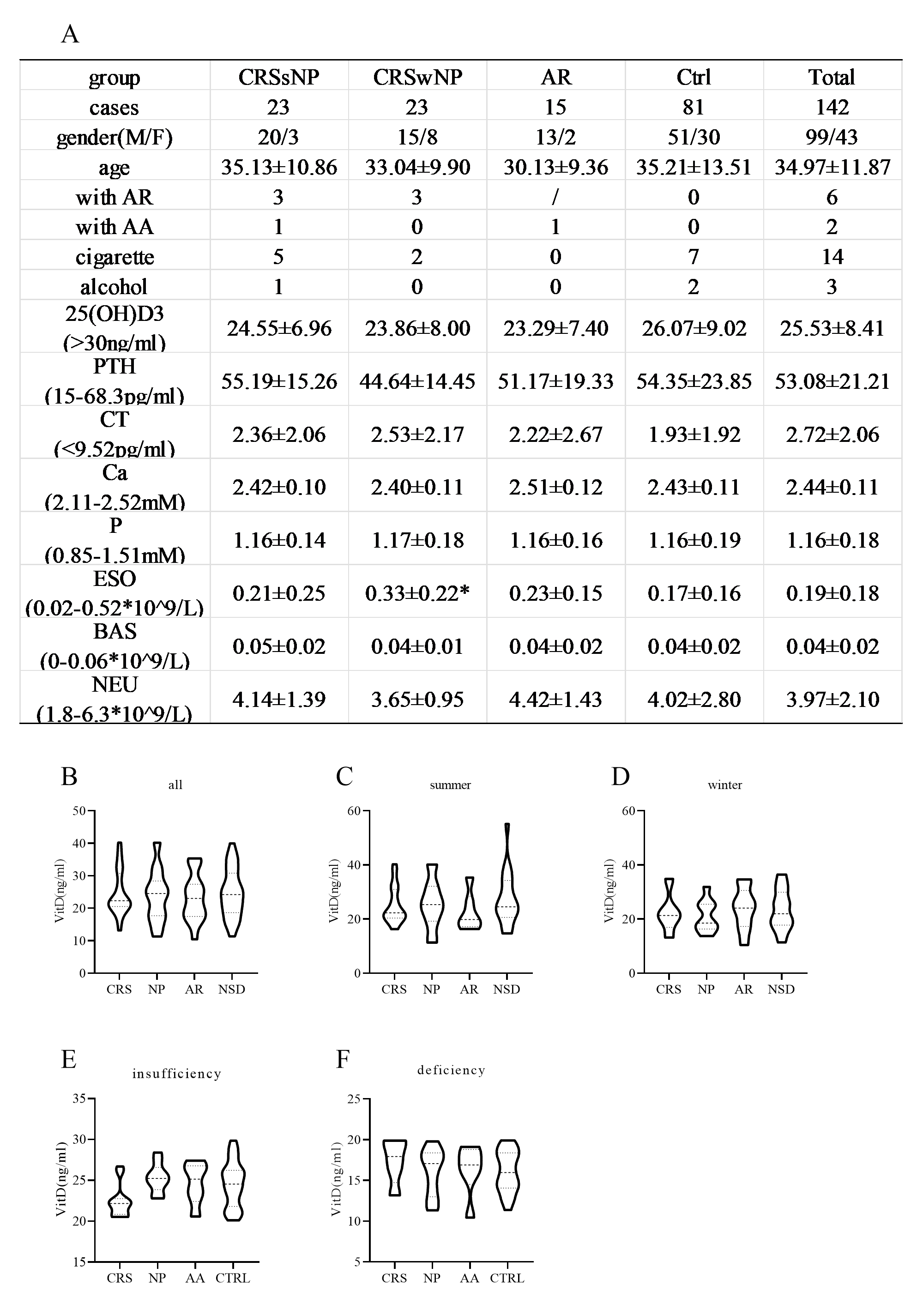

Supplement: Supplementary file 2 — Additional file 2: Figure S2. Clinical characteristics of enrolled cases. A 142 cases were enrolled and categorized into four groups as CRSsNP, CRSwNP, AR and Ctrl according to their prominent diagnosis, Ctrl group among which was composed of cases of patients without airway diseases as healthy control. Gender proportion, ageas well as cases complicated with AR and AA and cases with cigarette and alcohol consumption were recorded. The average levels of 25D3 were all below the least expected value of 30 ng/ml, either in subgroups or in total. Levels of PTH and CT, which are responsible for homeostasis of Ca and P, were both in normal ranges though levels of PTH were close to the upper limit. Levels of Ca and P were also within normal ranges. Levels of peripheral granulocytes including ESO, BAS and NEU were all among the normal ranges. Analysis of Variancesuggested each laboratory examination of the clinical indicators had no significant difference with the p > 0.05, except that EOS counts in CRSwNP group were higher than those in Ctrl group. B–F Violin plots of 25D3 concentration of all cases, cases in summer, cases in winter, cases in VD sufficiency and cases in VD deficiency, respectively, grouped by diseases, all showing a similar distribution of VD. CRSsNP chronic rhinosinusitis without nasal polyps, CRSwNP chronic rhinosinusitis with nasal polyps, AR allergic rhinitis, AA allergic asthma, M/f male/female, PTH parathyroid hormone, CT calcitonin, Ca serum calcium, P phosphate, EOS eosinophils, BAS basophils, NEU neutrophils. [file 12931_2023_2477_MOESM2_ESM.tif]
